# Non-Equilibrium Quantum Electrodynamics in Open Systems as a Realizable Representation of Quantum Field Theory of the Brain

**DOI:** 10.3390/e22010043

**Published:** 2019-12-27

**Authors:** Akihiro Nishiyama, Shigenori Tanaka, Jack A. Tuszynski

**Affiliations:** 1 Graduate School of System Informatics, Kobe University, 1-1 Rokkodai, Nada-ku, Kobe 657-8501, Japan; anishiyama@people.kobe-u.ac.jp (A.N.); tanaka2@kobe-u.ac.jp (S.T.); 2 Department of Oncology, University of Alberta, Cross Cancer Institute, Edmonton, AB T6G 1Z2, Canada; 3 Department of Physics, University of Alberta, Edmonton, AB T6G 2J1, Canada; 4 DIMEAS, Corso Duca degli Abruzzi, 24, Politecnico di Torino, 10129 Turin, Italy

**Keywords:** non-equilibrium quantum field theory, open systems, quantum electrodynamics, brain dynamics

## Abstract

We derive time evolution equations, namely the Klein–Gordon equations for coherent fields and the Kadanoff–Baym equations in quantum electrodynamics (QED) for open systems (with a central region and two reservoirs) as a practical model of quantum field theory of the brain. Next, we introduce a kinetic entropy current and show the H-theorem in the Hartree–Fock approximation with the leading-order (LO) tunneling variable expansion in the 1st order approximation for the gradient expansion. Finally, we find the total conserved energy and the potential energy for time evolution equations in a spatially homogeneous system. We derive the Josephson current due to quantum tunneling between neighbouring regions by starting with the two-particle irreducible effective action technique. As an example of potential applications, we can analyze microtubules coupled to a water battery surrounded by a biochemical energy supply. Our approach can be also applied to the information transfer between two coherent regions via microtubules or that in networks (the central region and the $N_{\mathrm{res}}$ reservoirs) with the presence of quantum tunneling.

## 1. Introduction

What is a physical mechanism of generating memory in the brain, and where is memory stored in the brain? These are still open questions in contemporary neuroscience [1,2]. We know that memory has an aspect of information encoding and retrieval as well meaning attached to this information. In information theory, we adopt Shannon entropy as a measure of information content [3]. This entropy increases as the uncertainty associated with information becomes larger. On the other hand, in thermodynamics we use thermodynamic Boltzmannian entropy as a measure of disorder in a physical system. This entropy increases as the order of the system is reduced. If Shannon entropy represents the same concept as thermodynamic entropy, we must adopt an ordered system to memorize information. There might be no way to memorize information without adopting an ordered physical system as has been earlier discussed within quantum field theory (QFT) [4]. In QFT, order is associated with the breakdown of symmetry [5]. For example, crystals are ordered quantum systems of discretely arranged atoms, where continuous translational symmetry is spontaneously broken. Order is maintained by long-range correlations involving phonons, with Nambu–Goldstone (NG) quanta [6,7,8] emerging as quantum excitations from the ground state in spontaneous symmetry breaking (SSB). Since NG quanta are massless, macroscopic order emerges due to these long-range correlations. Ferromagnets are examples of ordered quantum systems composed of magnetic dipoles aligned in the same direction, so that rotational symmetry is spontaneously broken when the ferromagnetic phase is generated by magnetic moment alignment. This order is maintained by long-range correlations involving magnons, which are massless NG quanta emerging in SSB. The concept of SSB can be adopted in QFT (with infinite unitarily- or physically-inequivalent vacua) distinguished from quantum mechanics in which the concept of SSB does not appear. Furthermore, QFT is conventionally applied to macroscopic matter, although its application is not restricted only to microscopic phenomena [5]. Hence, it is reasonable and convenient to adopt QFT with the possibility of spontaneous symmetry breakdown for the physical description of ordered systems, which contain information, or memory.

Quantum field theory of the brain, or quantum brain dynamics (QBD), represents a proposal to describe memory formation in the brain by adopting the breakdown of symmetry [9,10]. Memory in the brain has properties of heterogeneity, long-term but imperfect stability, and diffuse nonlocal nature Each memory is diffused and not localized to particular regions in the brain. It does not disappear due to the destruction of particular local regions. [11,12,13]. The QBD can describe these properties of memory in the brain by adopting unitarily inequivalent vacua, namely diverse coherent states. One vacuum is imperfectly stable and transferred to another over the course of time. Each vacuum is characterized by macroscopic spatial extension with long-range correlations. The QBD originated in the work by Ricciardi and Umezawa in 1967 [14], where external stimuli trigger SSB of the system or macroscopic order. In the 1970s, this model was further developed by Stuart et al. [15,16], whereby the brain is envisaged as a mixed system of classical neurons and microscopic degrees of freedom, namely corticons and exchange bosons, which were not specifically identified at this stage. Around the same time, Fröhlich studied a theory of biological coherence involving electric dipoles contained in the membranes of biological systems [17,18,19,20,21,22]. When the frequencies of oscillating dipoles in the system are within a narrow range around the resonance frequencies and coupling constants of interaction with heat bath and energy pump are large enough, an ordered state with the dielectric polarization (where electric dipoles are dynamically aligned in the same direction) emerges leading to the breakdown of symmetry and the coherent wave propagation of dipole oscillation forms a so-called Fröhlich condensate. In 1976, Davydov and Kislukha proposed a theory of solitary waves propagating in DNA and protein chains (alpha-helices) called the Davydov soliton [23]. The theories of coherence in biological systems by Fröhrich and Davydov can be described by static and dynamical properties of the nonlinear Schrödinger equation with an equivalent quantum Hamiltonian, respectively [24]. In the 1980s, Del Giudice et al., studied collective properties of electric dipoles of water in biological systems based on QFT [25,26,27]. After the analysis of water’s electric dipole fields in biological systems, Jibu and Yasue identified concrete microscopic degrees of freedom of QBD (corticons and exchange bosons) in the 1990s, namely water electric dipole fields and massive photon fields [9,28,29,30,31]. The QBD is essentially Quantum Electrodynamics (QED) of water electric dipoles. They adopted a superradiant phase, which represents the coherent state of water dipoles and massive photons [32,33,34,35,36]. When water electric dipoles are aligned in the same direction, the rotational symmetry is spontaneously broken, and polaritons, NG bosons, emerge in the SSB. They are absorbed into the longitudinal modes of photons, and photons acquire mass due to the Higgs mechanism. The massive photons are called evanescent photons. Since these photons have mass which is proportional to coherent dipole fields or the square root of the number density of aligned dipoles, they can stay in the dynamically-coherent regions of the brain. Memory in this model, therefore, is the coherent state of water electric dipole fields and photon fields with the condensation of the NG modes in the vacuum state. In 1995, Vitiello proposed a dissipative model of QBD to solve the problem of the informational capacity of memory [37]. As a result, a huge informational capacity of memory capacity was proposed to result by regarding the brain as an open system and by doubling the degrees of freedom. In 2003, Zheng and Pollack showed experimentally the existence of the so-called exclusion zone (EZ) water, which formed around hydrophilic surfaces [38] such as those around proteins. The properties of EZ water correspond to those of coherent water in QED [39].

However, the preceding research on this topic lacks the non-equilibrium multi-energy-mode analysis in open systems since it is based on the two-energy-level approximation for charged fields and single-energy-mode analysis for photon fields. Unsurprisingly, the main criticism found in the literature is related to the quantum decoherence phenomena, which means the formed coherent fields might rapidly disappear due to thermal effects destroying the broken symmetry states in the process [40]. Hence, memory proposed in the QFT models discussed above would be rapidly erased. In the above approximations, several components which might induce decoherence are lost, namely field-particle conversion (decoherence), thermal effects, and collision processes with multi-energy-mode incoherent particles. However, whether the decoherence occurs or not must be demonstrated by non-equilibrium numerical simulations based on the multi-energy-mode analysis in open systems. In case coherence is robust, we will be able to find non-equilibrium memory formation processes through numerical simulations.

The aim of this paper is to derive time evolution equations based on QED with charged bosons present in open systems in order to provide a theoretical framework for a concrete description of memory formation processes, which can be further developed in the future. In this paper, to describe multi-energy-mode phenomena, we adopt the Kadanoff–Baym (KB) equations [41,42,43] for quantum fluctuations in QED for open systems, with the use of Klein–Gordon (KG) equations for coherent fields. We can describe general dynamics with the above equations, since the Boltzmann equation, the hydrodynamic equations and the Langevin equations are derived from the KB equation [42,44,45]. We introduce a kinetic entropy current by use of the KB equations, and show the H-theorem in the Hartree–Fock approximation to the 1st order approximation in the gradient expansion. These approximations are adopted as coarse-graining procedures to define a kinetic entropy. We also find the total conserved charge and energy in spatially homogeneous systems. By use of the KG equations and the KB equations, we can describe non-equilibrium, non-secular, multi-energy-mode, charge-energy-conserving dynamics. Finally, we find that it is possible to describe the time evolution of gauge-invariant quantities. This is the main result of this paper.

This paper is organized as follows. In Section 2, we provide the Lagrangian density in QED in open systems (with a central region and two reservoirs coupled to it) and derive time evolution equations for coherent fields and quantum fluctuations. In Section 3, we analyze the Kadanoff–Baym equations and show the gauge invariance. In Section 4, we introduce a kinetic entropy current and show the H-theorem. In Section 5, we derive time evolution equations in spatially-homogeneous systems, and give the conserved charge and energy expression for total systems. In Section 6, we discuss our results. In Section 7, we provide conclusions derived from this work. In the Appendix A, several calculations for the $O(e^{4}|\bar{\varphi }{|}^{2})$ self-energy are given. In this paper, we adopt the metric $\eta^{\mu \nu }=\eta_{\mu \nu }=\mathrm{diag}\left(1,-1,-1,-1\right)$ in 3+1 dimensions where the Greek letters (μ,ν) run over 0 to *d* in d+1 dimensions and the subscripts (i,j) run over 1 to *d*. We use the Greek letter α to represent the left *L* and the right *R* reservoirs. The speed of light and the Planck constant divided by 2π are both set to be 1.

## 2. Two-Particle Irreducible Effective Action and Time Evolution Equations

In this section, we begin with the Lagrangian density of quantum electrodynamics (QED) with charged bosons in open systems, and derive time evolution equations for coherent fields and quantum fluctuations.

**Figure 1:**
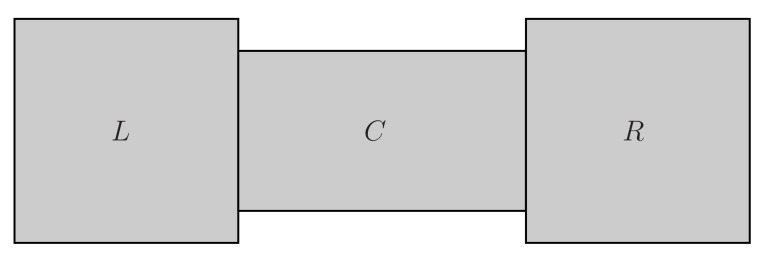
Open systems given by the central region *C* and the two reservoirs (*L* and *R*).

The Lagrangian density in open systems (the central region *C* and the two reservoirs L,R [46,47,48,49] with tunneling effects [50,51,52,53]) depicted in Figure 1 with the background field method [54,55,56,57] is given by,

(1) $$\begin{array}{c} L=L_{C}+\sum_{\alpha =L,R}L_{\alpha }+L_{\mathrm{tunnel}}. \end{array}$$

Here the Lagrangian density in the center region is

(2) $$\begin{array}{cc} L_{C} & =-\frac{1}{4}F_{\mu \nu }\left[A_{C}+a_{C}\right]F^{\mu \nu }\left[A_{C}+a_{C}\right]\\ & +\left[\left(\partial_{\mu }+ie\left(A_{C,\mu }+a_{C,\mu }\right)\right)\varphi_{C}^{*}\right]\left[\left(\partial^{\mu }-ie\left(A_{C}^{\mu }+a_{C}^{\mu }\right)\right)\varphi_{C}\right]-m^{2}\varphi_{C}^{*}\varphi_{C}\\ & -\frac{1}{2\xi }{\left(\partial_{\mu }a_{C}^{\mu }\right)}^{2}, \end{array}$$

where $A_{C}^{\mu }$ is the background photon field in *C*, $a_{C}^{\mu }$ represents the quantum fluctuations in *C*, $F_{\mu \nu }\left[A_{C}\right]=\partial_{\mu }A_{C,\nu }-\partial_{\nu }A_{C,\mu }$, $\varphi_{C}^{*}$ and $\varphi_{C}$ are charged Bose fields in *C*, *m* is the mass of the charged bosons. The Lagrangian density $L_{\alpha }$ in α=L and *R* is given by changing the labels *C* in fields in Equation (2) to α, namely by $A_{\alpha }^{\mu }$, $a_{\alpha }^{\mu }$, $\varphi_{\alpha }^{*}$ and $\varphi_{\alpha }$. The tunneling Lagrangian density $L_{\mathrm{tunnel}}$ is

(3) $$\begin{array}{c} L_{\mathrm{tunnel}}=+\sum_{\alpha =L,R}\left(v_{\alpha }\left(x\right)\varphi_{C}^{*}\left(x\right)\varphi_{\alpha }\left(x\right)+v_{\alpha }^{*}\left(x\right)\varphi_{\alpha }^{*}\left(x\right)\varphi_{C}\left(x\right)+v_{a,\alpha }a_{C,i}\left(x\right)a_{\alpha ,i}\left(x\right)\right), \end{array}$$

where we have introduced the tunneling variables $v_{\alpha }\left(x\right)$ of charged bosons and the tunneling coupling $v_{a,\alpha }$ of photons. We find that the total Lagrangian density in Equation (1) is invariant under the Type I gauge transformations [54,55,56,57] in open systems, that is,

(4) $$\begin{array}{c} \varphi_{C}\to e^{i\chi_{C}}\varphi_{C},\;\;\;\varphi_{C}^{*}\to e^{-i\chi_{C}}\varphi_{C}^{*},\;\;\;A_{C}^{\mu }\to A_{C}^{\mu }+\frac{1}{e}\partial^{\mu }\chi_{C},\;\;\;a_{C}^{\mu }\to a_{C}^{\mu },\\ \varphi_{\alpha }\to e^{i\chi_{\alpha }}\varphi_{\alpha },\;\;\;\varphi_{\alpha }^{*}\to e^{-i\chi_{\alpha }}\varphi_{\alpha }^{*},\;\;\;A_{\alpha }^{\mu }\to A_{\alpha }^{\mu }+\frac{1}{e}\partial^{\mu }\chi_{\alpha },\;\;\;a_{\alpha }^{\mu }\to a_{\alpha }^{\mu },\\ v_{\alpha }\to v_{\alpha }e^{i(\chi_{C}-\chi_{\alpha })}. \end{array}$$

**Figure 2:**
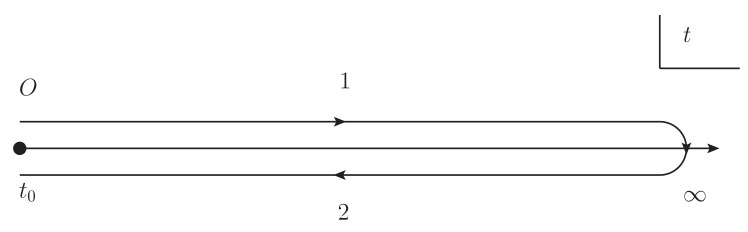
Closed-time-path contour C. The label 1 represents the path from $t_{0}$ to *∞*, and the label 2 represents the path from *∞* to $t_{0}$.

To describe non-equilibrium processes, we consider quantum fields in the closed-time-path C (the Keldysh contour) with the path 1 from $t_{0}$ to *∞* and the path 2 from *∞* to $t_{0}$ depicted in Figure 2. We impose gauge fixing conditions $a_{C}^{0}=0$, and $a_{\alpha }^{0}=0$ with α=L,R on the generating functional with the above total Lagrangian in Equation (1). We adopt the functional integral with $a_{C}$, $a_{\alpha }$, $\varphi_{C}$, $\varphi_{C}^{*}$, $\varphi_{\alpha }$, and $\varphi_{\alpha }^{*}$ with α=L,R in the generating functional. We shall perform the Legendre transformation of the generating functional. Then we can derive two-particle irreducible (2PI) effective action $\Gamma_{2\mathrm{PI}}\left[A,{\bar{a}}^{i}=0,\bar{\varphi },{\bar{\varphi }}^{*},\Delta ,D\right]$ as

(5) $$\begin{array}{cc} \Gamma_{2\mathrm{PI}}\left[A,{\bar{a}}^{i}=0,\bar{\varphi },{\bar{\varphi }}^{*},\Delta ,D\right] & =\int_{C}d^{d+1}x[-\frac{1}{4}F_{\mu \nu }\left[A_{C}\right]F^{\mu \nu }\left[A_{C}\right]\\ & +\left[\left(\partial_{\mu }+ieA_{C,\mu }\right){\bar{\varphi }}_{C}^{*}\right]\left[\left(\partial^{\mu }-ieA_{C}^{\mu }\right){\bar{\varphi }}_{C}\right]-m^{2}{\bar{\varphi }}_{C}^{*}{\bar{\varphi }}_{C}\\ & +(C\to \alpha =L,\;R)\\ & +\sum_{\alpha =L,R}\left(v_{\alpha }\left(x\right){\bar{\varphi }}_{C}^{*}{\bar{\varphi }}_{\alpha }+v_{\alpha }^{*}\left(x\right){\bar{\varphi }}_{\alpha }^{*}{\bar{\varphi }}_{C}\right)]\\ & +\frac{i}{2}\mathrm{Tr}\ln D^{-1}+\frac{i}{2}\mathrm{Tr}D_{0}^{-1}D+i\mathrm{Tr}\ln\Delta^{-1}+i\mathrm{Tr}\Delta_{0}^{-1}\Delta \\ & +\frac{1}{2}\Gamma_{2}\left[A,\bar{\varphi },{\bar{\varphi }}^{*},\Delta ,D\right], \end{array}$$

where *d* is the spatial dimensions, $\bar{\varphi }\equiv \langle \varphi^{}\rangle$, ${\bar{\varphi }}^{*}\equiv \langle \varphi^{*}\rangle$, and ${\bar{a}}^{i}\equiv \langle a^{i}\rangle$ with brackets 〈·〉≡Tr(densitymatrix)×(·). The $i\Delta_{0}^{-1}$ is given by a 3×3 matrix as

(6) $$\begin{array}{cc} i\Delta_{0}^{-1}\left(x,y\right)= & \frac{\delta^{2}\int d^{d+1}zL\left(z\right)}{\delta \varphi^{*}\left(x\right)\delta \varphi \left(y\right)}{|}_{a_{C}=a_{\alpha }=0}\\ = & \left[\begin{array}{ccc} i\Delta_{0,LL}^{-1}\left(x,y\right) & v_{L}\left(x\right)\delta_{C}^{d+1}\left(x-y\right) & 0\\ v_{L}^{*}\left(x\right)\delta_{C}^{d+1}\left(x-y\right) & i\Delta_{0,CC}^{-1}\left(x,y\right) & v_{R}^{*}\left(x\right)\delta_{C}^{d+1}\left(x-y\right)\\ 0 & v_{R}\left(x\right)\delta_{C}^{d+1}\left(x-y\right) & i\Delta_{0,RR}^{-1}\left(x,y\right) \end{array}\right], \end{array}$$

with

(7) $$\begin{array}{c} i\Delta_{0,\alpha \alpha }^{-1}\left(x,y\right)=\left[-\partial_{x}^{2}+ie\left(A_{\alpha ,\mu }\left(y\right)\partial_{y}^{\mu }-A_{\alpha ,\mu }\left(x\right)\partial_{x}^{\mu }\right)+e^{2}A_{\alpha }^{\mu }\left(x\right)A_{\alpha ,\mu }\left(x\right)-m^{2}\right]\delta_{C}^{d+1}\left(x-y\right), \end{array}$$

(α=L,R), and

(8) $$\begin{array}{c} i\Delta_{0,CC}^{-1}\left(x,y\right)=\left[-\partial_{x}^{2}+ie\left(A_{C,\mu }\left(y\right)\partial_{y}^{\mu }-A_{C,\mu }\left(x\right)\partial_{x}^{\mu }\right)+e^{2}A_{C}^{\mu }\left(x\right)A_{C,\mu }\left(x\right)-m^{2}\right]\delta_{C}^{d+1}\left(x-y\right). \end{array}$$

Further, $iD_{0}^{-1}$ is given by a 3×3 matrix as

(9) $$\begin{array}{cc} iD_{0,ij}^{-1}\left(x,y\right)= & \frac{\delta^{2}\int d^{d+1}zL\left(z\right)}{\delta a^{i}\left(x\right)\delta a^{j}\left(y\right)}{|}_{{\bar{a}}_{C}={\bar{a}}_{\alpha }=0}\\ = & \left[\begin{array}{ccc} iD_{0,LL,ij}^{-1}\left(x,y\right) & v_{a,L}\delta_{ij}\delta_{C}^{d+1}\left(x-y\right) & 0\\ v_{a,L}\delta_{ij}\delta_{C}^{d+1}\left(x-y\right) & iD_{0,CC,ij}^{-1}\left(x,y\right) & v_{a,R}\delta_{ij}\delta_{C}^{d+1}\left(x-y\right)\\ 0 & v_{a,R}\delta_{ij}\delta_{C}^{d+1}\left(x-y\right) & iD_{0,RR,ij}^{-1}\left(x,y\right) \end{array}\right], \end{array}$$

where

(10) $$\begin{array}{ccc} iD_{0,\alpha \alpha ,ij}^{-1}\left(x,y\right) & = & \left(-\partial_{x}^{2}-2e^{2}{\bar{\varphi }}_{\alpha }^{*}{\bar{\varphi }}_{\alpha }\right)\delta_{ij}\delta_{C}^{d+1}\left(x-y\right),\;\;\;\left(\alpha =L,R\right) \end{array}$$

(11) $$\begin{array}{ccc} iD_{0,CC,ij}^{-1}\left(x,y\right) & = & \left(-\partial_{x}^{2}-2e^{2}{\bar{\varphi }}_{C}^{*}{\bar{\varphi }}_{C}\right)\delta_{ij}\delta_{C}^{d+1}\left(x-y\right), \end{array}$$

and we set the gauge fixing parameter as ξ=1.

The Green function Δ(x,y) is written by a 3×3 matrix as

(12) $$\begin{array}{cc} \Delta (x,y)= & \left[\begin{array}{ccc} \Delta_{LL}\left(x,y\right) & \Delta_{LC}\left(x,y\right) & \Delta_{LR}\left(x,y\right)\\ \Delta_{CL}\left(x,y\right) & \Delta_{CC}\left(x,y\right) & \Delta_{CR}\left(x,y\right)\\ \Delta_{RL}\left(x,y\right) & \Delta_{RC}\left(x,y\right) & \Delta_{RR}\left(x,y\right) \end{array}\right]\\ = & \left[\begin{array}{ccc} \langle T_{C}\delta \varphi_{L}^{*}\left(x\right)\delta \varphi_{L}\left(y\right)\rangle & \langle T_{C}\delta \varphi_{L}^{*}\left(x\right)\delta \varphi_{C}\left(y\right)\rangle & \langle T_{C}\delta \varphi_{L}^{*}\left(x\right)\delta \varphi_{R}\left(y\right)\rangle \\ \langle T_{C}\delta \varphi_{C}^{*}\left(x\right)\delta \varphi_{L}\left(y\right)\rangle & \langle T_{C}\delta \varphi_{C}^{*}\left(x\right)\delta \varphi_{C}\left(y\right)\rangle & \langle T_{C}\delta \varphi_{C}^{*}\left(x\right)\delta \varphi_{R}\left(y\right)\rangle \\ \langle T_{C}\delta \varphi_{R}^{*}\left(x\right)\delta \varphi_{L}\left(y\right)\rangle & \langle T_{C}\delta \varphi_{R}^{*}\left(x\right)\delta \varphi_{C}\left(y\right)\rangle & \langle T_{C}\delta \varphi_{R}^{*}\left(x\right)\delta \varphi_{R}\left(y\right)\rangle \end{array}\right], \end{array}$$

with $\delta \varphi =\varphi -\bar{\varphi }$, and $T_{C}$ representing the time-ordered product in the closed-time-path C. It is possible to express each component in the above matrix by a 2×2 matrix in the closed-time-path,

(13) $$\begin{array}{c} \Delta_{LC}\left(x,y\right)=\left[\begin{array}{cc} \Delta_{LC}^{11}\left(x,y\right) & \Delta_{LC}^{12}\left(x,y\right)\\ \Delta_{LC}^{21}\left(x,y\right) & \Delta_{LC}^{22}\left(x,y\right) \end{array}\right]=\left[\begin{array}{cc} \langle T\delta \varphi_{L}^{*}\left(x\right)\delta \varphi_{C}\left(y\right)\rangle & \langle \delta \varphi_{C}\left(y\right)\delta \varphi_{L}^{*}\left(x\right)\rangle \\ \langle \delta \varphi_{L}^{*}\left(x\right)\delta \varphi_{C}\left(y\right)\rangle & \langle \tilde{T}\delta \varphi_{L}^{*}\left(x\right)\delta \varphi_{C}\left(y\right)\rangle \end{array}\right], \end{array}$$

with *T* representing the time-ordered product, and $\tilde{T}$ representing the anti-time-ordered product.

Similarly, the Green function $D_{ij}\left(x,y\right)$ is written as a 3×3 matrix as

(14) $$\begin{array}{cc} D_{ij}\left(x,y\right) & =\left[\begin{array}{ccc} D_{LL,ij}\left(x,y\right) & D_{LC,ij}\left(x,y\right) & D_{LR,ij}\left(x,y\right)\\ D_{CL,ij}\left(x,y\right) & D_{CC,ij}\left(x,y\right) & D_{CR,ij}\left(x,y\right)\\ D_{RL,ij}\left(x,y\right) & D_{RC,ij}\left(x,y\right) & D_{RR,ij}\left(x,y\right) \end{array}\right], \end{array}$$

where $D_{CL,ij}\left(x,y\right)=\langle T_{C}a_{C,i}\left(x\right)a_{L,j}\left(y\right)\rangle$, and $D_{00}\left(x,y\right)=D_{0i}\left(x,y\right)=D_{i0}\left(x,y\right)=0$ with i=1,···d.

The following relations for the 2PI effective action $\Gamma_{2\mathrm{PI}}$ are derived using the Legendre transformation,

(15) $$\begin{array}{c} \frac{\delta \Gamma_{2\mathrm{PI}}}{\delta \Delta }=0,\;\;\;\frac{\delta \Gamma_{2\mathrm{PI}}}{\delta D}=0, \end{array}$$

and

(16) $$\begin{array}{ccc} \frac{\delta \Gamma_{2\mathrm{PI}}}{\delta a^{i}}{|}_{{\bar{a}}^{i}=0}=\frac{\delta \Gamma_{2\mathrm{PI}}}{\delta A^{i}}{|}_{{\bar{a}}^{i}=0}=0, & \frac{\delta \Gamma_{2\mathrm{PI}}}{\delta {\bar{\varphi }}^{*}}=0, & \frac{\delta \Gamma_{2\mathrm{PI}}}{\delta \bar{\varphi }}=0, \end{array}$$

where the coherent fields are labeled by *C* or α=L,R. By use of the relations in Equation (15), we can derive,

(17) $$\begin{array}{c} i\left(\Delta_{0}^{-1}-\tilde{\Sigma }\right)=i\Delta^{-1}, \end{array}$$

and

(18) $$\begin{array}{c} i\left(D_{0}^{-1}-\Pi \right)=iD^{-1}, \end{array}$$

with the definition of self-energy, $i\tilde{\Sigma }\equiv -\frac{1}{2}\frac{\delta \Gamma_{2}}{\delta \Delta }$ and $i\Pi \equiv -\frac{\delta \Gamma_{2}}{\delta D}$. The self-energy is given by,

(19) $$\begin{array}{c} \tilde{\Sigma }\left(x,y\right)=\left[\begin{array}{ccc} {\tilde{\Sigma }}_{LL}\left(x,y\right) & 0 & 0\\ 0 & {\tilde{\Sigma }}_{CC}\left(x,y\right) & 0\\ 0 & 0 & {\tilde{\Sigma }}_{RR}\left(x,y\right) \end{array}\right], \end{array}$$

and

(20) $$\begin{array}{c} \Pi_{ij}\left(x,y\right)=\left[\begin{array}{ccc} \Pi_{LL,ij}\left(x,y\right) & 0 & 0\\ 0 & \Pi_{CC,ij}\left(x,y\right) & 0\\ 0 & 0 & \Pi_{RR,ij}\left(x,y\right) \end{array}\right]. \end{array}$$

We neglect off-diagonal elements, since they represent a higher order of the tunneling variables or the tunneling coupling constants. The explicit forms of diagonal elements are given by labeling CC or αα with α=L,R in Green functions of self-energy in [58]. These relations are the Kadanoff–Baym (KB) equations in open systems.

Next we derive the Klein–Gordon (KG) equations for coherent fields. The first equation in Equation (16) is written by,

(21) $$\begin{array}{ccc} \partial^{\nu }F_{\nu i}\left[A_{C}\left(x\right)\right] & = & J_{C,i}, \end{array}$$

(22) $$\begin{array}{ccc} \partial^{\nu }F_{\nu i}\left[A_{\alpha }\left(x\right)\right] & = & J_{\alpha ,i}, \end{array}$$

where we define

(23) $$\begin{array}{cc} J_{C,\mu } & \equiv eJ_{C,\mu }-ie\left[\left(\partial_{i}^{x_{1}}-ieA_{C,i}\left(x_{1}\right)\right)\Delta_{CC}^{11}\left(x,x_{1}\right){|}_{x_{1}=x}-\left(\partial_{i}^{x_{2}}+ieA_{C,i}\left(x_{2}\right)\right)\Delta_{CC}^{11}\left(x_{2},x\right){|}_{x_{2}=x}\right]\\ & -\frac{1}{2}\frac{\delta \Gamma_{2}}{\delta A_{C}^{\mu }\left(x\right)}, \end{array}$$

and

(24) $$\begin{array}{cc} J_{\alpha ,\mu } & \equiv eJ_{\alpha ,\mu }-ie\left[\left(\partial_{i}^{x_{1}}-ieA_{\alpha ,i}\left(x_{1}\right)\right)\Delta_{\alpha \alpha }^{11}\left(x,x_{1}\right){|}_{x_{1}=x}-\left(\partial_{i}^{x_{2}}+ieA_{\alpha ,i}\left(x_{2}\right)\right)\Delta_{\alpha \alpha }^{11}\left(x_{2},x\right){|}_{x_{2}=x}\right]\\ & -\frac{1}{2}\frac{\delta \Gamma_{2}}{\delta A_{\alpha }^{\mu }\left(x\right)}, \end{array}$$

where $J_{C,\mu }\equiv i\left[-{\bar{\varphi }}_{C}^{*}\left(\partial_{\mu }-ieA_{C,\mu }\right){\bar{\varphi }}_{C}+\left(\left(\partial_{\mu }+ieA_{C,\mu }\right){\bar{\varphi }}_{C}^{*}\right){\bar{\varphi }}_{C}\right]$ and $J_{\alpha ,\mu }\equiv i\left[-{\bar{\varphi }}_{\alpha }^{*}\left(\partial_{\mu }-ieA_{\alpha ,\mu }\right){\bar{\varphi }}_{\alpha }+\left(\left(\partial_{\mu }+ieA_{\alpha ,\mu }\right){\bar{\varphi }}_{\alpha }^{*}\right){\bar{\varphi }}_{\alpha }\right]$. Here $\frac{\delta \Gamma_{2}}{\delta A_{C}^{0}\left(x\right)}=\frac{\delta \Gamma_{2}}{\delta A_{\alpha }^{0}\left(x\right)}=0$ due to the gauge fixing condition $a_{C}^{0}=a_{\alpha }^{0}=0$.

The second and the third equations in Equation (16) are written by

(25) $$\begin{array}{c} -\left(\partial_{\mu }-ieA_{C,\mu }\right)\left(\partial^{\mu }-ieA_{C}^{\mu }\right){\bar{\varphi }}_{C}-m^{2}{\bar{\varphi }}_{C}-e^{2}D_{CC,ii}^{11}\left(x,x\right){\bar{\varphi }}_{C}+\sum_{\alpha =L,R}v_{\alpha }\left(x\right){\bar{\varphi }}_{\alpha }\left(x\right)+\frac{1}{2}\frac{\delta \Gamma_{2}}{\delta {\bar{\varphi }}_{C}^{*}}=0, \end{array}$$

(26) $$\begin{array}{c} -\left(\partial_{\mu }+ieA_{C,\mu }\right)\left(\partial^{\mu }+ieA_{C}^{\mu }\right){\bar{\varphi }}_{C}^{*}-m^{2}{\bar{\varphi }}_{C}^{*}-e^{2}D_{CC,ii}^{11}\left(x,x\right){\bar{\varphi }}_{C}^{*}+\sum_{\alpha =L,R}v_{\alpha }^{*}\left(x\right){\bar{\varphi }}_{\alpha }^{*}\left(x\right)+\frac{1}{2}\frac{\delta \Gamma_{2}}{\delta {\bar{\varphi }}_{C}}=0, \end{array}$$

(27) $$\begin{array}{c} -\left(\partial_{\mu }-ieA_{\alpha ,\mu }\right)\left(\partial^{\mu }-ieA_{\alpha }^{\mu }\right){\bar{\varphi }}_{\alpha }-m^{2}{\bar{\varphi }}_{\alpha }-e^{2}D_{\alpha \alpha ,ii}^{11}\left(x,x\right){\bar{\varphi }}_{\alpha }+v_{\alpha }^{*}\left(x\right){\bar{\varphi }}_{C}\left(x\right)+\frac{1}{2}\frac{\delta \Gamma_{2}}{\delta {\bar{\varphi }}_{\alpha }^{*}}=0, \end{array}$$

(28) $$\begin{array}{c} -\left(\partial_{\mu }+ieA_{\alpha ,\mu }\right)\left(\partial^{\mu }+ieA_{\alpha }^{\mu }\right){\bar{\varphi }}_{\alpha }^{*}-m^{2}{\bar{\varphi }}_{\alpha }^{*}-e^{2}D_{\alpha \alpha ,ii}^{11}\left(x,x\right){\bar{\varphi }}_{\alpha }^{*}+v_{\alpha }\left(x\right){\bar{\varphi }}_{C}^{*}\left(x\right)+\frac{1}{2}\frac{\delta \Gamma_{2}}{\delta {\bar{\varphi }}_{\alpha }}=0. \end{array}$$

By using the above four equations and the Kadanoff–Baym equations in Equation (17), we can derive the total charge conservation

(29) $$\begin{array}{c} \partial^{\mu }\left(J_{C,\mu }+\sum_{\alpha =L,R}J_{\alpha ,\mu }\right)=0, \end{array}$$

in the Hartree–Fock approximation in the coupling expansion, in $\frac{1}{2}\Gamma_{2}$ in [58] and to the leading-order (LO) in the tunneling coupling expansion for the KG and the KB equations. Using the total charge conservation, the identity $\partial^{\mu }\partial^{\nu }F_{\mu \nu }=0$, and Equations (21) and (22), we arrive at,

(30) $$\begin{array}{cc} \partial^{0}\left(J_{C,0}+\sum_{\alpha =L,R}J_{\alpha ,0}\right) & =-\partial^{i}\left(J_{C,i}+\sum_{\alpha =L,R}J_{\alpha ,i}\right)\\ & =-\partial^{\nu }\partial^{i}\left[F_{\nu i}\left[A_{C}\right]+\sum_{\alpha =L,R}F_{\nu i}\left[A_{\alpha }\right]\right]\\ & =\partial^{\nu }\partial^{\mu }F_{\nu \mu }\left[A_{C}+\sum_{\alpha }A_{\alpha }\right]-\partial^{\nu }\partial^{i}F_{\nu i}\left[A_{C}+\sum_{\alpha }A_{\alpha }\right]\\ & =\partial^{\nu }\partial^{0}F_{\nu 0}\left[A_{C}+\sum_{\alpha }A_{\alpha }\right], \end{array}$$

namely,

(31) $$\begin{array}{c} \partial^{\nu }F_{\nu 0}\left[A_{C}+\sum_{\alpha }A_{\alpha =L,R}\right]=J_{C,0}+\sum_{\alpha =L,R}J_{\alpha ,0}. \end{array}$$

Here, the time-independent term in the time integration which might be interpreted as an initial condition is set to be zero.

## 3. The Kadanoff–Baym Equations in QED in Open Systems

In this section, we write the Kadanoff–Baym (KB) equations in QED in open systems to the 1st order approximation in the gradient expansion by introducing gauge-invariant Green functions under Type I gauge transformation in Equation (4). We find that time evolution equations in diagonal elements are written only by gauge-invariant functions to the 1st order in the gradient expansion. We use the α=L,R to represent the two reservoirs to avoid confusing ‘*R*’ (Retarded in Green functions and self-energy) and ‘*L*’ (Longitudinal modes in Green functions and self-energy for photons) in this section. We set $t_{0}$ to −∞.

We begin with the KB equations given in the previous section. We multiply the matrix Δ from the right in Equation (17) and take the (C,C) component, then we write,

(32) $$\begin{array}{c} \left[i\left(\Delta_{0,CC}^{-1}-{\tilde{\Sigma }}_{CC}\right)\Delta_{CC}\right]\left(x,y\right)+\sum_{\alpha }v_{\alpha }^{*}\left(x\right)\Delta_{\alpha C}\left(x,y\right)=i\delta_{C}\left(x-y\right). \end{array}$$

We define

(33) $$\begin{array}{c} I_{C}\left(x,y\right)\equiv e\int_{y}^{x}dz_{\mu }A_{C}^{\mu }\left(z\right). \end{array}$$

We then multiply Equation (32) by $\exp\left(iI_{C}\left(x,y\right)\right)$ [59,60] and define the gauge-invariant Green function and gauge-invariant self-energy as

(34) $$\begin{array}{ccc} G_{CC}\left(x,y\right) & \equiv & \exp\left(iI_{C}\left(x,y\right)\right)\Delta_{CC}\left(x,y\right), \end{array}$$

(35) $$\begin{array}{ccc} \Sigma_{CC}\left(x,y\right) & \equiv & \exp\left(iI_{C}\left(x,y\right)\right){\tilde{\Sigma }}_{CC}\left(x,y\right), \end{array}$$

under the Type I gauge transformation in Equation (4). We next Fourier-transform by the relative coordinate x−y with $\int d\left(x-y\right)e^{ip\cdot (x-y)}$ and neglect terms beyond than 1st order in the gradient expansion in Equation (32), then we know that the 1st term on the left-hand side in Equation (32) can be written by gauge-invariant functions in the 1st order in the gradient expansion [58,61,62,63,64,65]. We show that the 2nd term on the left-hand side in Equation (32) is invariant under the Type I gauge transformation in Equation (4) in the 1st order in the gradient expansion. The (α,C) component in Equation (17) multiplied by the matrix Δ from the right is written as

(36) $$\begin{array}{c} \left[i\left(\Delta_{0,\alpha \alpha }^{-1}-{\tilde{\Sigma }}_{\alpha \alpha }\right)\Delta_{\alpha C}\right]\left(w,y\right)+v_{\alpha }\left(w\right)\Delta_{CC}\left(w,y\right)=0. \end{array}$$

Here, it is convenient to define the function $\Delta_{g,\alpha \alpha }^{-1}$ satisfying

(37) $$\begin{array}{c} i\Delta_{g,\alpha \alpha }^{-1}=i\left(\Delta_{0,\alpha \alpha }^{-1}-{\tilde{\Sigma }}_{\alpha \alpha }\right). \end{array}$$

Using Equation (37) in Equation (36), we arrive at

(38) $$\begin{array}{c} \Delta_{\alpha C}\left(x,y\right)=-\frac{1}{i}\int_{C,w}\Delta_{\alpha \alpha }\left(x,w\right)v_{\alpha }\left(w\right)\Delta_{CC}\left(w,y\right). \end{array}$$

When we define

(39) $$\begin{array}{c} I_{\alpha }\left(x,y\right)\equiv e\int_{y}^{x}dz_{\mu }A_{\alpha }^{\mu }\left(z\right), \end{array}$$

(40) $$\begin{array}{c} \phi_{C,yxw}\equiv I_{C}\left(y,w\right)+I_{C}\left(x,y\right)+I_{C}\left(w,x\right)∼e\int dS_{\mu \nu }F^{\mu \nu }, \end{array}$$

(with the Stokes theorem and the surface integral $\int dS^{\mu \nu }$ of the triangle yxw) and

(41) $$\begin{array}{c} g_{\alpha \alpha }\left(x,y\right)\equiv \exp\left(iI_{\alpha }\left(x,y\right)\right)\Delta_{g,\alpha \alpha }\left(x,y\right), \end{array}$$

we arrive at

(42) $$\begin{array}{cc} v_{\alpha }^{*}\left(x\right)\exp\left(iI_{C}\left(x,y\right)\right)\Delta_{\alpha C}\left(x,y\right) & =-\frac{1}{i}\int_{C,w}v_{\alpha }^{*}\left(x\right)e^{i\left(\phi_{C,yxw}+I_{C}\left(x,w\right)+I_{C}\left(w,y\right)\right)}\Delta_{g,\alpha \alpha }\left(x,w\right)\\ & \;\times e^{i\left(I_{\alpha }\left(x,w\right)-I_{\alpha }\left(x,w\right)\right)}v_{\alpha }\left(w\right)\Delta_{CC}\left(w,y\right)\\ & =-\frac{1}{i}\int_{C,w}e^{i\phi_{C,yxw}}V_{\alpha }\left(x,w\right)g_{\alpha \alpha }\left(x,w\right)G_{CC}\left(w,y\right), \end{array}$$

with the definition

(43) $$\begin{array}{c} V_{\alpha }\left(x,w\right)\equiv v_{\alpha }^{*}\left(x\right)\exp\left(iI_{C}\left(x,w\right)-iI_{\alpha }\left(x,w\right)\right)v_{\alpha }\left(w\right). \end{array}$$

We find $V_{\alpha }\left(x,w\right)$ is gauge invariant under the Type I gauge transformation in Equation (4). Later, we show $g_{\alpha \alpha }$ is a gauge-invariant function in the 1st order in the gradient expansion. With the use of Equation (42), the Fourier transformation of Equation (32) by $\int_{x-y}e^{ip\cdot (x-y)}$ after multiplying $e^{iI_{C}}$ in the matrix notation is rewritten as,

(44) $$\begin{array}{c} i\left(G_{0}^{-1}1-\Sigma_{CC}\sigma_{z}\right)\circ_{C}G_{CC}\left(X,p\right)+i\sum_{\alpha }\int_{x-y}e^{ip\cdot (x-y)}\int_{w}e^{i\phi_{C,yxw}}V_{\alpha }\left(x,w\right)g_{\alpha \alpha }\left(x,w\right)\sigma_{z}G_{CC}\left(w,y\right)=i, \end{array}$$

where $X=\frac{x+y}{2}$, $iG_{0}^{-1}\left(p\right)=\left(p^{2}-m^{2}\right)$, 1=diag(1,1), and $\sigma_{z}=\mathrm{diag}\left(1,-1\right)$. Here, we have used the Moyal product [61,62,63,64,65] in QED in open systems. When we neglect terms beyond the 1st order of the gradient expansion in the Moyal product, we find that

(45) $$\begin{array}{c} M\circ_{C}N=M\left(X,p\right)N\left(X,p\right)+\frac{i}{2}{\left\{M,N\right\}}_{C}+O\left(\frac{\partial^{2}}{\partial X^{2}}\right), \end{array}$$

with the arbitrary function M(X,p) and N(X,p) and the Poisson bracket in *C* written as

(46) $$\begin{array}{c} {\left\{M,N\right\}}_{C}=\frac{\partial M}{\partial p^{\mu }}\frac{\partial N}{\partial X_{\mu }}-\frac{\partial M}{\partial X^{\mu }}\frac{\partial N}{\partial p_{\mu }}-eE_{C}\cdot \left(\frac{\partial M}{\partial p}\frac{\partial N}{\partial p^{0}}-\frac{\partial M}{\partial p^{0}}\frac{\partial N}{\partial p}\right)+eB_{C}\cdot \left(\frac{\partial M}{\partial p}\times \frac{\partial N}{\partial p}\right), \end{array}$$

where the electric field E and the magnetic field B in the central region *C* are introduced. The electromagnetic fields appear by expanding $e^{i\phi_{C,yxw}}$ in the convolution integral. In Equation (44), we define

(47) $$\begin{array}{c} U_{\alpha \alpha }\left(x,w\right)\equiv V_{\alpha }\left(x,w\right)g_{\alpha \alpha }\left(x,w\right), \end{array}$$

to arrive at

(48) $$\begin{array}{c} i\left(G_{0}^{-1}1-\Sigma_{CC}\sigma_{z}+\sum_{\alpha }U_{\alpha \alpha }\sigma_{z}\right)\circ_{C}G_{CC}=i\sigma_{z}, \end{array}$$

where $U_{\alpha \alpha }$ is a function of (X,p). The functions $G_{0}^{-1}\left(p\right)$, $G_{CC}\left(X,p\right)$, $\Sigma_{CC}\left(X,p\right)$, $g_{\alpha \alpha }\left(X,p\right)$, and $\sigma_{z}$ are written using a 2×2 matrix in the closed-time-path, but the Fourier-transformed $V_{\alpha }\left(X,p\right)$ is a scalar function.

Next, we multiply the matrix Δ from the left in Equation (17) and take the (C,C) and (C,α) component. Then we can write

(49) $$\begin{array}{c} \left[\Delta_{CC}\left(i\Delta_{0,CC}^{-1}-i{\tilde{\Sigma }}_{CC}\right)\right]\left(x,y\right)+\sum_{\alpha }\Delta_{C\alpha }\left(x,y\right)v_{\alpha }\left(y\right)=i\delta_{C}\left(x-y\right), \end{array}$$

and

(50) $$\begin{array}{c} \left[\Delta_{C\alpha }\left(i\Delta_{0,\alpha \alpha }^{-1}-i{\tilde{\Sigma }}_{\alpha \alpha }\right)\right]\left(x,w\right)+\Delta_{CC}\left(x,w\right)v_{\alpha }^{*}\left(w\right)=0. \end{array}$$

With the use of Equations (37) and (50), we can write

(51) $$\begin{array}{c} \Delta_{C\alpha }\left(x,y\right)=-\frac{1}{i}\Delta_{CC}\left(x,w\right)v_{\alpha }^{*}\left(w\right)\Delta_{g,\alpha \alpha }\left(w,y\right), \end{array}$$

and taking into account Equations (33), (39), (40), and definitions (34), (41) and (43),

(52) $$\begin{array}{cc} e^{iI_{C}\left(x,y\right)}\Delta_{C\alpha }\left(x,y\right)v_{\alpha }\left(y\right) & =\int_{C,w}ie^{iI_{C}\left(w,y\right)+iI_{C}\left(x,w\right)+i\phi_{C,yxw}}\Delta_{CC}\left(x,w\right)v_{\alpha }^{*}\left(w\right)\Delta_{g,\alpha \alpha }\left(w,y\right)v_{\alpha }\left(y\right)\\ & \;\times e^{iI_{\alpha }\left(w,y\right)-iI_{\alpha }\left(w,y\right)}\\ & =\int_{C,w}e^{i\phi_{C,yxw}}G_{CC}\left(x,w\right)g_{\alpha \alpha }\left(w,y\right)v_{\alpha }^{*}\left(w\right)e^{iI_{C}\left(w,y\right)-iI_{\alpha }\left(w,y\right)}v_{\alpha }\left(y\right)\\ & =\int_{C,w}G_{CC}\left(x,w\right)g_{\alpha \alpha }\left(w,y\right)V_{\alpha }\left(w,y\right)e^{i\phi_{C,yxw}}. \end{array}$$

With the use of the above equation, we can rewrite Equation (49) in the matrix notation after the Fourier transformation:

(53) $$\begin{array}{c} G_{CC}\circ_{C}\left(i1G_{0}^{-1}-i\sigma_{z}\Sigma_{CC}+i\sigma_{z}\sum_{\alpha }U_{\alpha \alpha }\right)=i\sigma_{z}, \end{array}$$

where Green functions, self-energy and $U_{\alpha \alpha }$ are functions of (X,p) and a 2×2 matrix in the closed-time-path.

It is possible to derive the solution of the retarded Green function $G_{CC,R}\equiv i\left(G_{CC}^{11}-G_{CC}^{12}\right)$ of the 0th and 1st order equations in the gradient expansion. We rewrite the self-energy as $\Sigma_{CC}\left(x,y\right)=-i\delta_{C}\left(x-y\right)\Sigma_{CC,\mathrm{loc}}\left(x\right)+\Sigma_{CC,\mathrm{nonl}}\left(x,y\right)$ and use the Fourier transformation of the self-energy. By summing Equations (48) and (53) and taking the difference of (1,1) and (1,2) components, we arrive at,

(54) $$\begin{array}{c} \left(iG_{0}^{-1}\left(p\right)-\Sigma_{CC,\mathrm{loc}}\left(X\right)-\Sigma_{CC,R}\left(X,p\right)+\sum_{\alpha }U_{\alpha \alpha ,R}\left(X,p\right)\right)G_{CC,R}\left(X,p\right)=-1, \end{array}$$

where the retarded functions are defined as $\Sigma_{CC,R}\equiv i\left(\Sigma_{CC,\mathrm{nonl}}^{11}-\Sigma_{CC,\mathrm{nonl}}^{12}\right)$ and $U_{\alpha \alpha ,R}\equiv i\left(U_{\alpha \alpha }^{11}-U_{\alpha \alpha }^{12}\right)$. Furthermore, by taking the difference of Equations (48) and (53) and taking the difference of (a,b)=(1,1) and (1,2) components, we arrive at,

(55) $$\begin{array}{c} {\left\{iG_{0}^{-1}\left(p\right)-\Sigma_{CC,\mathrm{loc}}\left(X\right)-\Sigma_{CC,R}\left(X,p\right)+\sum_{\alpha }U_{\alpha \alpha ,R}\left(X,p\right),G_{CC,R}\left(X,p\right)\right\}}_{C}=0. \end{array}$$

The solution of the above two equations is

(56) $$\begin{array}{c} G_{CC,R}=\frac{-1}{p^{2}-m^{2}-\Sigma_{\mathrm{loc}}-\Sigma_{CC,R}+\sum_{\alpha }U_{\alpha \alpha }}. \end{array}$$

The spectral function defined as $\rho_{CC}\left(X,p\right)\equiv i\left(G_{CC}^{21}-G_{CC}^{12}\right)$ is given by taking the imaginary part of $G_{CC,R}$ and multiplying by 2.

Next, with the help of the relation (37), we can derive an expression for $g_{\alpha \alpha }$ as

(57) $$\begin{array}{c} \left(iG_{0}^{-1}1-i\Sigma_{\alpha \alpha }\sigma_{z}\right)\circ_{\alpha }g_{\alpha \alpha }=i\sigma_{z}, \end{array}$$

and

(58) $$\begin{array}{c} g_{\alpha \alpha }\circ_{\alpha }\left(i1G_{0}^{-1}-i\sigma_{z}\Sigma_{\alpha \alpha }\right)=i\sigma_{z}, \end{array}$$

in the matrix notation in the closed-time-path. Here the Moyal product $\circ_{\alpha }$ in the reservoir α represents

(59) $$\begin{array}{c} M\circ_{\alpha }N=M\left(X,p\right)N\left(X,p\right)+\frac{i}{2}{\left\{M,N\right\}}_{\alpha }+O\left(\frac{\partial^{2}}{\partial X^{2}}\right), \end{array}$$

with the arbitrary function M(X,p) and N(X,p) and the Poisson bracket in α written as

(60) $$\begin{array}{c} {\left\{M,N\right\}}_{\alpha }=\frac{\partial M}{\partial p^{\mu }}\frac{\partial N}{\partial X_{\mu }}-\frac{\partial M}{\partial X^{\mu }}\frac{\partial N}{\partial p_{\mu }}-eE_{\alpha }\cdot \left(\frac{\partial M}{\partial p}\frac{\partial N}{\partial p^{0}}-\frac{\partial M}{\partial p^{0}}\frac{\partial N}{\partial p}\right)+eB_{\alpha }\cdot \left(\frac{\partial M}{\partial p}\times \frac{\partial N}{\partial p}\right). \end{array}$$

We derive the solution of the retarded Green function $g_{\alpha \alpha ,R}\equiv i\left(g_{\alpha \alpha }^{11}-g_{\alpha \alpha }^{12}\right)$

(61) $$\begin{array}{c} g_{\alpha \alpha ,R}=\frac{-1}{p^{2}-m^{2}-\Sigma_{\alpha \alpha ,\mathrm{loc}}-\Sigma_{\alpha \alpha ,R}}, \end{array}$$

where we rewrite the self-energy as $\Sigma_{\alpha \alpha }\left(x,y\right)=-i\delta_{C}\left(x-y\right)\Sigma_{\alpha \alpha ,\mathrm{loc}}\left(x\right)+\Sigma_{\alpha \alpha ,\mathrm{nonl}}\left(x,y\right)$ and define $\Sigma_{\alpha \alpha ,R}\equiv i\left(\Sigma_{\alpha \alpha ,\mathrm{nonl}}^{11}-\Sigma_{\alpha \alpha ,\mathrm{nonl}}^{12}\right)$.

Next we derive time evolution equations of the (α,α) components. Let us multiply the matrix Δ from the right in Equation (17) and take the (α,α) components. Then we can write them as

(62) $$\begin{array}{c} \left[i\left(\Delta_{0,\alpha \alpha }^{-1}-{\tilde{\Sigma }}_{\alpha \alpha }\right)\Delta_{\alpha \alpha }\right]\left(x,y\right)+v_{\alpha }\left(x\right)\Delta_{C\alpha }\left(x,y\right)=i\delta_{C}\left(x-y\right). \end{array}$$

We know that the 1st term in the above equation is written by gauge-invariant functions given by

(63) $$\begin{array}{ccc} G_{\alpha \alpha }\left(x,y\right) & \equiv & \exp\left(iI_{\alpha }\left(x,y\right)\right)\Delta_{\alpha \alpha }\left(x,y\right), \end{array}$$

(64) $$\begin{array}{ccc} \Sigma_{\alpha \alpha }\left(x,y\right) & \equiv & \exp\left(iI_{\alpha }\left(x,y\right)\right){\tilde{\Sigma }}_{\alpha \alpha }\left(x,y\right), \end{array}$$

by multiplying the link variable $e^{iI_{\alpha }\left(x,y\right)}$ in the same way as in the isolated system [58,65]. Taking into account Equation (51) and the definition

(65) $$\begin{array}{c} \phi_{\alpha ,yxw}\equiv I_{\alpha }\left(y,w\right)+I_{\alpha }\left(x,y\right)+I_{\alpha }\left(w,x\right), \end{array}$$

we can write

(66) $$\begin{array}{cc} e^{iI_{\alpha }\left(x,y\right)}v_{\alpha }\left(x\right)\Delta_{C\alpha }\left(x,y\right) & =\int_{C,w}e^{iI_{\alpha }\left(x,y\right)}v_{\alpha }\left(x\right)i\Delta_{CC}\left(x,w\right)v_{\alpha }^{*}\left(w\right)\Delta_{g,\alpha \alpha }\left(w,y\right)\\ & =\int_{C,w}e^{iI_{\alpha }\left(x,y\right)}v_{\alpha }\left(x\right)i\Delta_{CC}\left(x,w\right)e^{iI_{C}\left(x,w\right)+iI_{C}\left(w,x\right)}v_{\alpha }^{*}\left(w\right)\\ & \;\times \Delta_{g,\alpha \alpha }\left(w,y\right)e^{iI_{\alpha }\left(w,y\right)+iI_{\alpha }\left(y,w\right)}\\ & =\int_{C,w}e^{iI_{\alpha }\left(x,y\right)}v_{\alpha }\left(x\right)iG_{CC}\left(x,w\right)e^{iI_{C}\left(w,x\right)}v_{\alpha }^{*}\left(w\right)g_{\alpha \alpha }\left(w,y\right)e^{iI_{\alpha }\left(y,w\right)}\\ & =\int_{C,w}iG_{CC}\left(x,w\right)g\left(w,y\right)v_{\alpha }\left(x\right)e^{iI_{C}\left(w,x\right)-iI_{\alpha }\left(w,x\right)}v_{\alpha }^{*}\left(w\right)e^{i\phi_{\alpha ,yxw}}\\ & =\int_{C,w}iG_{CC}\left(x,w\right)V_{\alpha }\left(w,x\right)g\left(w,y\right)e^{i\phi_{\alpha ,yxw}}, \end{array}$$

with the definitions (34), (41) and (43). Using the definition

(67) $$\begin{array}{c} Q_{\alpha \alpha }\left(x,w\right)\equiv G_{CC}\left(x,w\right)V_{\alpha }\left(w,x\right), \end{array}$$

Equation (62) is written after the Fourier transformation with $\int_{x-y}e^{ip\cdot (x-y)}$ as

(68) $$\begin{array}{c} \left(iG_{0}^{-1}1-i\Sigma_{\alpha \alpha }\sigma_{z}\right)\circ_{\alpha }G_{\alpha \alpha }+iQ_{\alpha \alpha }\sigma_{z}\circ_{\alpha }g_{\alpha \alpha }=i\sigma_{z}. \end{array}$$

Multiplying the matrix Δ from the left in Equation (17) and taking the (α,α) components, we arrive at

(69) $$\begin{array}{c} G_{\alpha \alpha }\circ_{\alpha }\left(i1G_{0}^{-1}-i\sigma_{z}\Sigma_{\alpha \alpha }\right)+ig_{\alpha \alpha }\circ_{\alpha }\sigma_{z}Q_{\alpha \alpha }=i\sigma_{z}. \end{array}$$

In a similar way to [66], the 0th order solution of the retarded Green function $G_{\alpha \alpha ,R}\equiv i\left(G_{\alpha ,\alpha }^{11}-G_{\alpha \alpha }^{12}\right)$ is derived as

(70) $$\begin{array}{c} G_{\alpha \alpha ,R}=g_{\alpha \alpha ,R}+g_{\alpha \alpha ,R}Q_{\alpha \alpha ,R}g_{\alpha \alpha ,R}, \end{array}$$

with $Q_{\alpha \alpha ,R}\equiv i\left(Q_{\alpha \alpha }^{11}-Q_{\alpha \alpha }^{12}\right)$. It is derived by multiplying Equation (62) by $\Delta_{g,\alpha \alpha }$ from the left, multiplying $e^{iI_{\alpha }}$, and taking only the 0th order terms in the difference of (1,1) and (1,2) components after the Fourier transformation. It is also the solution of the 1st order equation of the retarded Green function written by

(71) $$\begin{array}{c} {\left\{iG_{0}^{-1}-\Sigma_{\alpha \alpha ,\mathrm{loc}}-\Sigma_{\alpha \alpha ,R},G_{\alpha \alpha ,R}\right\}}_{\alpha }+{\left\{Q_{\alpha \alpha ,R},g_{\alpha \alpha ,R}\right\}}_{\alpha }=0, \end{array}$$

which is derived by taking the difference of Equations (68) and (69) and taking the difference of (1,1) and (1,2) components. The spectral function $\rho_{\alpha \alpha }\equiv i\left(G_{\alpha \alpha }^{21}-G_{\alpha \alpha }^{12}\right)$ is given by taking the imaginary part in Equation (70) and multiplying by 2.

Next, we comment on the gauge dependence of $g_{\alpha \alpha }$. The relation (37) means that the gauge-dependent function $\Delta_{g,\alpha \alpha }^{-1}$ has the same gauge dependence as that on the right-hand side. The explicit form of $\Delta_{0,\alpha \alpha }^{-1}$ is given in Equation (7), and the explicit form of self-energy ${\tilde{\Sigma }}_{\alpha \alpha }$ in $O(e^{2})$ and $O(e^{4}|\bar{\varphi }{|}^{2})$ is calculated in the same way as the case in the isolated system [58] or the Appendix A. We then show that $H\equiv e^{iI_{\alpha }}\Delta_{g,\alpha \alpha }^{-1}=e^{iI_{\alpha }}\left(\Delta_{0,\alpha \alpha }^{-1}-{\tilde{\Sigma }}_{\alpha \alpha }\right)$ is written in gauge-invariant form as in the isolated system [65]. But no explicit gauge dependence of $\Delta_{g,\alpha \alpha }$ is given, although the explicit gauge dependence of $\Delta_{CC}$ and $\Delta_{\alpha \alpha }$ is given in their definition. We then multiply $\Delta_{g,\alpha \alpha }$, the inverse function of $\Delta_{g,\alpha \alpha }^{-1}$, in Equation (37) from left and right and multiply $e^{iI_{\alpha }}$, and then take the Fourier transformation to arrive at

(72) $$\begin{array}{c} H\circ g=1,\;\;\;\;g\circ H=1, \end{array}$$

where $g\equiv e^{iI_{\alpha }}\Delta_{g}$ and we omit the label αα and the label α in the Moyal product. We now perform Type I gauge transformation for Equation (37) and repeat the same procedures as above. Since *H* is gauge invariant, we can write gauge transformed $g_{h}$ as

(73) $$\begin{array}{c} H\circ g_{h}=1,\;\;\;\;g_{h}\circ H=1. \end{array}$$

If there is gauge dependence $g_{h}=g+\epsilon$, the above two relations impose

(74) $$\begin{array}{c} H\circ \epsilon =0,\;\;\;\;\epsilon \circ H=0. \end{array}$$

We can write the solution of the retarded $\epsilon_{R}$ in Equation (74) in the 0th and 1st order in the gradient expansion in the same way as [58,67]

(75) $$\begin{array}{c} \epsilon_{R}=\frac{\zeta }{p^{2}-m^{2}-\Sigma_{\mathrm{loc},\alpha \alpha }-\Sigma_{R,\alpha \alpha }}, \end{array}$$

with ζ=0 since the right-hand side in Equation (74) is zero. Since the charged bosons are massive (no infrared divergence), m≠0, and the spectral width ImΣR,αα(X,p) in $\Gamma_{2}\neq 0$ around $p^{0}=\pm \sqrt{p^{2}+m^{2}+\cdot \cdot \cdot }$ is nonzero (even if much smaller than $m^{2}$) in the presence of nonzero continuous particle distributions in (X,p) in general, the solution is $\epsilon_{R}\left(X,p\right)=0$ (no peaks) and ϵ(x,y)=0. Hence, even if the gauge dependence in *g* remains, it is of higher order in the gradient expansion.

Finally, we write the Kadanoff–Baym equations for photons. Starting with Equation (18), we can derive

(76) $$\begin{array}{ccc} {\left(iD_{0,CC}^{-1}1-i\Pi_{CC}\sigma_{z}\right)}_{ik}\circ D_{CC,kj}+i\sum_{\alpha }v_{a,\alpha }^{2}d_{\alpha \alpha ,ik}\sigma_{z}\circ D_{CC,kj} & = & i\sigma_{z}\delta_{ij}, \end{array}$$

(77) $$\begin{array}{ccc} D_{CC,ik}\circ {\left(i1D_{0,CC}^{-1}-i\sigma_{z}\Pi_{CC}\right)}_{kj}+i\sum_{\alpha }v_{a,\alpha }^{2}D_{CC,ik}\circ \sigma_{z}d_{\alpha \alpha ,kj} & = & i\sigma_{z}\delta_{ij}, \end{array}$$

with $iD_{0,CC,ik}^{-1}\left(X,p\right)=\left(p^{2}-2e^{2}{\bar{\varphi }}_{C}^{*}\left(X\right){\bar{\varphi }}_{C}\left(X\right)\right)\delta_{ik}$ and

(78) $$\begin{array}{ccc} {\left(iD_{0}^{-1}1-i\Pi_{\alpha \alpha }\sigma_{z}\right)}_{ik}\circ d_{\alpha \alpha ,kj} & = & i\sigma_{z}\delta_{ij}, \end{array}$$

(79) $$\begin{array}{ccc} d_{\alpha \alpha ,ik}\circ {\left(i1D_{0}^{-1}-i\sigma_{z}\Pi_{\alpha \alpha }\right)}_{kj} & = & i\sigma_{z}\delta_{ij}. \end{array}$$

Here, the Moyal product ∘ for photons to the 1st order in the gradient expansion is

(80) $$\begin{array}{c} M\circ N=M\left(X,p\right)N\left(X,p\right)+\frac{i}{2}\left\{M,N\right\}+O\left(\frac{\partial^{2}}{\partial X^{2}}\right), \end{array}$$

with

(81) $$\begin{array}{c} \left\{M,N\right\}=\frac{\partial M}{\partial p^{\mu }}\frac{\partial N}{\partial X_{\mu }}-\frac{\partial M}{\partial X^{\mu }}\frac{\partial N}{\partial p_{\mu }}. \end{array}$$

The (α,α) components are

(82) $$\begin{array}{ccc} {\left(iD_{0,\alpha \alpha }^{-1}1-i\Pi_{\alpha \alpha }\sigma_{z}\right)}_{ik}\circ D_{\alpha \alpha ,kj}+iv_{a,\alpha }^{2}D_{CC,ik}\circ \sigma_{z}d_{\alpha \alpha ,kj} & = & i\sigma_{z}\delta_{ij}, \end{array}$$

(83) $$\begin{array}{ccc} D_{\alpha \alpha ,ik}\circ {\left(i1D_{0,\alpha \alpha }^{-1}-i\sigma_{z}\Pi_{\alpha \alpha }\right)}_{kj}+iv_{a,\alpha }^{2}d_{\alpha \alpha ,ik}\sigma_{z}\circ D_{CC,kj} & = & i\sigma_{z}\delta_{ij}, \end{array}$$

with $iD_{0,\alpha \alpha ,ik}^{-1}\left(X,p\right)=\left(p^{2}-2e^{2}{\bar{\varphi }}_{\alpha }^{*}\left(X\right){\bar{\varphi }}_{\alpha }\left(X\right)\right)\delta_{ik}$.

## 4. Kinetic Entropy Current and the H-Theorem

In this section, we derive a kinetic entropy current in QED in open systems by adopting the 1st order approximation in the gradient expansion as a coarse-graining procedure [67,68,69,70,71], and show the H-theorem in the Hartree–Fock approximation in the coupling expansion and in the leading-order (LO) approximation of the tunneling variable and tunneling coupling expansion. In this section, we use the α=L,R to represent the two reservoirs, the *R* represents the ‘retarded’ and the *L* represents the longitudinal of photons.

If we subtract Equation (53) from Equation (48), then we can derive

(84) $$\begin{array}{cc} i{\left\{p^{2}-m^{2}-\Sigma_{CC,\mathrm{loc}},G_{CC}^{ab}\right\}}_{C} & =i{\left[\left(\Sigma_{CC,\mathrm{nonl}}-\sum_{\alpha }U_{\alpha \alpha }\right)\sigma_{z}\circ_{C}G_{CC}\right]}^{ab}\\ & \;-i{\left[G_{CC}\circ_{C}\sigma_{z}\left(\Sigma_{CC,\mathrm{nonl}}-\sum_{\alpha }U_{\alpha \alpha }\right)\right]}^{ab}. \end{array}$$

We find that this equation has the same form as [58] with changing $\Sigma_{\mathrm{nonl}}$ to $\Sigma_{CC,\mathrm{nonl}}-\sum_{\alpha }U_{\alpha \alpha }$ in deriving the kinetic entropy current. By using the Kadanoff–Baym Ansatz $G_{CC}^{12}=\frac{\rho_{CC}}{i}f_{CC}$, $G_{CC}^{21}=\frac{\rho_{CC}}{i}\left(1+f_{CC}\right)$, $\Sigma_{CC}^{12}=\frac{\Sigma_{CC,\rho }}{i}\gamma_{CC}$, $\Sigma_{CC}^{21}=\frac{\Sigma_{CC,\rho }}{i}\left(1+\gamma_{CC}\right)$, $U_{\alpha \alpha }^{12}=\frac{U_{\alpha \alpha ,\rho }}{i}\gamma_{U,\alpha \alpha }$ and $U_{\alpha \alpha }^{21}=\frac{U_{\alpha \alpha ,\rho }}{i}\left(1+\gamma_{U,\alpha \alpha }\right)$ with $\Sigma_{CC,\rho }\equiv i\left(\Sigma_{CC}^{21}-\Sigma_{CC}^{12}\right)$ and $U_{\alpha \alpha ,\rho }\equiv i\left(U_{\alpha \alpha }^{21}-U_{\alpha \alpha }^{12}\right)$, and by neglecting the 2nd order terms in the gradient expansion [67,68,69,70,71], we can use

(85) $$\begin{array}{c} f_{CC}∼\gamma_{CC}∼\gamma_{U,\alpha \alpha }. \end{array}$$

Then we arrive at

(86) $$\begin{array}{cc} \partial_{\mu }s_{\mathrm{matter},C}^{\mu } & =-\int_{p}\left(\Sigma_{CC,\mathrm{nonl}}^{21}G_{CC}^{12}-\Sigma_{CC,\mathrm{nonl}}^{12}G_{CC}^{21}\right)\ln\frac{G_{CC}^{12}}{G_{CC}^{21}}\\ & \;+\sum_{\alpha }\int_{p}\left(U_{\alpha \alpha }^{21}G_{CC}^{12}-U_{\alpha \alpha }^{12}G_{CC}^{21}\right)\ln\frac{G_{CC}^{12}}{G_{CC}^{21}}, \end{array}$$

with

(87) smatter,Cμ=2[pμ−12∂Re(ΣCC,R−∑αUαα,R)∂pμρCCi +12∂ReGCC,R∂pμΣCC,ρ−∑αUαα,ρi]σ[fCC],

and

(88) $$\begin{array}{c} \sigma \left[f\right]=\left((1+f)\ln(1+f)-f\ln f\right). \end{array}$$

We subtract Equation (69) from Equation (68), to find

(89) $$\begin{array}{cc} i{\left\{p^{2}-m^{2}-\Sigma_{\alpha \alpha ,\mathrm{loc}},G_{\alpha \alpha }^{ab}\right\}}_{\alpha } & =i{\left[\Sigma_{\alpha \alpha ,\mathrm{nonl}}\sigma_{z}\circ_{\alpha }G_{\alpha \alpha }\right]}^{ab}-i{\left[G_{\alpha \alpha }\circ_{\alpha }\sigma_{z}\Sigma_{\alpha \alpha ,\mathrm{nonl}}\right]}^{ab}\\ & \;-i{\left[Q_{\alpha \alpha }\sigma_{z}\circ_{\alpha }g_{\alpha \alpha }\right]}^{ab}+i{\left[g_{\alpha \alpha }\circ_{\alpha }\sigma_{z}Q_{\alpha \alpha }\right]}^{ab}. \end{array}$$

By using the Kadanoff–Baym Ansatz $G_{\alpha \alpha }^{12}=\frac{\rho_{\alpha \alpha }}{i}f_{\alpha \alpha }$, $G_{\alpha \alpha }^{21}=\frac{\rho_{\alpha \alpha }}{i}\left(1+f_{\alpha \alpha }\right)$, $\Sigma_{\alpha \alpha }^{12}=\frac{\Sigma_{\alpha \alpha ,\rho }}{i}\gamma_{\alpha \alpha }$, $\Sigma_{\alpha \alpha }^{21}=\frac{\Sigma_{\alpha \alpha ,\rho }}{i}\left(1+\gamma_{\alpha \alpha }\right)$, $Q_{\alpha \alpha }^{12}=\frac{Q_{\alpha \alpha ,\rho }}{i}\gamma_{Q,\alpha \alpha }$ and $Q_{\alpha \alpha }^{21}=\frac{Q_{\alpha \alpha ,\rho }}{i}\left(1+\gamma_{Q,\alpha \alpha }\right)$ with $\Sigma_{\alpha \alpha ,\rho }\equiv i\left(\Sigma_{\alpha \alpha }^{21}-\Sigma_{\alpha \alpha }^{12}\right)$ and $Q_{\alpha \alpha ,\rho }\equiv i\left(Q_{\alpha \alpha }^{21}-Q_{\alpha \alpha }^{12}\right)$, and neglecting the 2nd order terms in the gradient expansion, we use

(90) $$\begin{array}{c} f_{\alpha \alpha }∼\gamma_{\alpha \alpha },\;\;\;\gamma_{g,\alpha \alpha }∼\gamma_{Q,\alpha \alpha }. \end{array}$$

We can also use

(91) $$\begin{array}{c} \gamma_{g,\alpha \alpha }∼\gamma_{\alpha \alpha }, \end{array}$$

by neglecting the 2nd order terms in the gradient expansion in the difference of Equation (57) and Equation (58) written by

(92) $$\begin{array}{ccc} i{\left\{p^{2}-m^{2}-\Sigma_{\alpha \alpha ,\mathrm{loc}},g_{\alpha \alpha }^{ab}\right\}}_{\alpha } & = & i{\left[\Sigma_{\alpha \alpha ,\mathrm{nonl}}\sigma_{z}\circ_{\alpha }g_{\alpha \alpha }\right]}^{ab}-i{\left[g_{\alpha \alpha }\circ_{\alpha }\sigma_{z}\Sigma_{\alpha \alpha ,\mathrm{nonl}}\right]}^{ab} \end{array}$$

Then, we obtain the following relation in a similar way as [66].

(93) $$\begin{array}{cc} \partial_{\mu }s_{\mathrm{matter},\alpha }^{\mu } & =-\int_{p}\left(\Sigma_{\alpha \alpha ,\mathrm{nonl}}^{21}G_{\alpha \alpha }^{12}-\Sigma_{\alpha \alpha ,\mathrm{nonl}}^{12}G_{\alpha \alpha }^{21}\right)\ln\frac{G_{\alpha \alpha }^{12}}{G_{\alpha \alpha }^{21}}\\ & \;+\int_{p}\left(Q_{\alpha \alpha }^{21}g_{\alpha \alpha }^{12}-Q_{\alpha \alpha }^{12}g_{\alpha \alpha }^{21}\right)\ln\frac{G_{\alpha \alpha }^{12}}{G_{\alpha \alpha }^{21}}, \end{array}$$

with

(94) smatter,αμ=2[pμ−12∂ReΣαα,R∂pμρααi+12∂ReGαα,R∂pμΣαα,ρi +12∂ReQαα,R∂pμgαα,ρi−∂Regαα,R∂pμQαα,ρi]σ[fαα].

We find that for $s_{\mathrm{matter}}^{\mu }=s_{\mathrm{matter},C}^{\mu }+\sum_{\alpha }s_{\mathrm{matter},\alpha }^{\mu }$

(95) $$\begin{array}{cc} \partial_{\mu }s_{\mathrm{matter}}^{\mu } & =-\int_{p}\left(\Sigma_{CC,\mathrm{nonl}}^{21}G_{CC}^{12}-\Sigma_{CC,\mathrm{nonl}}^{12}G_{CC}^{21}\right)\ln\frac{G_{CC}^{12}}{G_{CC}^{21}}\\ & \;-\sum_{\alpha }\int_{p}\left(\Sigma_{\alpha \alpha ,\mathrm{nonl}}^{21}G_{\alpha \alpha }^{12}-\Sigma_{\alpha \alpha ,\mathrm{nonl}}^{12}G_{\alpha \alpha }^{21}\right)\ln\frac{G_{\alpha \alpha }^{12}}{G_{\alpha \alpha }^{21}}\\ & \;+\sum_{\alpha }\int_{p}\left(U_{\alpha \alpha }^{21}G_{CC}^{12}-U_{\alpha \alpha }^{12}G_{CC}^{21}\right)\ln\frac{G_{CC}^{12}}{G_{CC}^{21}}\\ & \;+\sum_{\alpha }\int_{p}\left(Q_{\alpha \alpha }^{21}g_{\alpha \alpha }^{12}-Q_{\alpha \alpha }^{12}g_{\alpha \alpha }^{21}\right)\ln\frac{G_{\alpha \alpha }^{12}}{G_{\alpha \alpha }^{21}}. \end{array}$$

We show that the 3rd and 4th term on the right-hand side in the above equation is positive definite. Using the definitions $U_{\alpha \alpha }\left(x,w\right)\equiv V_{\alpha }\left(x,w\right)g_{\alpha \alpha }\left(x,w\right)$ and $Q_{\alpha \alpha }\left(x,w\right)\equiv G_{CC}\left(x,w\right)V_{\alpha }\left(w,x\right)$, we can re-express them after the Fourier transformation as

(96) $$\begin{array}{ccc} U_{\alpha \alpha }^{ab}\left(X,p\right) & = & \int_{k}V_{\alpha }\left(X,k\right)g_{\alpha \alpha }^{ab}\left(X,p-k\right), \end{array}$$

(97) $$\begin{array}{ccc} Q_{\alpha \alpha }^{ab}\left(X,p\right) & = & \int_{k}V_{\alpha }\left(X,k\right)G_{CC}^{ab}\left(X,p+k\right), \end{array}$$

with $X=\frac{x+w}{2}$ and the real function

(98) $$\begin{array}{cc} V_{\alpha }\left(X,k\right) & =\int_{x-w}e^{ik\cdot (x-w)}v_{\alpha }^{*}\left(x\right)e^{iI_{C}\left(x,w\right)-iI_{\alpha }\left(x,w\right)}v_{\alpha }\left(w\right)\\ & =\int_{z}e^{ik\cdot z}v_{\alpha }^{*}\left(X+\frac{z}{2}\right)e^{iI_{C}\left(X+\frac{z}{2},X-\frac{z}{2}\right)-iI_{\alpha }\left(X+\frac{z}{2},X-\frac{z}{2}\right)}v_{\alpha }\left(X-\frac{z}{2}\right)\\ & =\int_{z}e^{ik\cdot z}\left|v_{\alpha }\left(X+\frac{z}{2}\right)\right|e^{-i\left(\beta_{C}\left(X+\frac{z}{2}\right)-\beta_{\alpha }\left(X+\frac{z}{2}\right)+\theta_{\alpha }\left(X+\frac{z}{2}\right)\right)}\\ & \;\times e^{iI_{C}\left(X+\frac{z}{2},X-\frac{z}{2}\right)-iI_{\alpha }\left(X+\frac{z}{2},X-\frac{z}{2}\right)}\left|v_{\alpha }\left(X-\frac{z}{2}\right)\right|e^{i\left(\beta_{C}\left(X-\frac{z}{2}\right)-\beta_{\alpha }\left(X-\frac{z}{2}\right)+\theta_{\alpha }\left(X-\frac{z}{2}\right)\right)}\\ & ∼\int_{z}e^{ik\cdot z}{\left|v_{\alpha }\right|}^{2}e^{i\left(e\left(A_{C}-\frac{\partial \beta_{C}}{e}\right)-e\left(A_{\alpha }-\frac{\partial \beta_{\alpha }}{e}\right)-\partial \theta_{\alpha }\right)\cdot z}+O\left(\frac{\partial^{2}}{\partial X^{2}}\right)\\ & =|v_{\alpha }{|}^{2}{\left(2\pi \right)}^{d+1}\delta^{d+1}\left(k+e\left(A_{C}-\frac{\partial \beta_{C}}{e}\right)-e\left(A_{\alpha }-\frac{\partial \beta_{\alpha }}{e}\right)-\partial \theta_{\alpha }\right), \end{array}$$

where we have used the expansion $v_{\alpha }=\left|v_{\alpha }\right|e^{i(\beta_{C}-\beta_{\alpha }+\theta_{\alpha })}$ with ${\bar{\varphi }}_{C}=\left|{\bar{\varphi }}_{C}\right|e^{i\beta_{C}}$ and ${\bar{\varphi }}_{\alpha }=\left|{\bar{\varphi }}_{\alpha }\right|e^{i\beta_{\alpha }}$. Here, the $A_{C}-\frac{\partial \beta_{C}}{e}$ and the $A_{\alpha }-\frac{\partial \beta_{\alpha }}{e}$ are invariant under the Type I gauge transformation, and these physical quantities are introduced in a similar way to [72]. We find that $V_{\alpha }\left(k\right)$ is a semi-positive definite in the 1st order in the gradient expansion. Then, we arrive at

(99) $$\begin{array}{c} +\sum_{\alpha }\int_{p}\left(U_{\alpha \alpha }^{21}G_{CC}^{12}-U_{\alpha \alpha }^{12}G_{CC}^{21}\right)\ln\frac{G_{CC}^{12}}{G_{CC}^{21}}+\sum_{\alpha }\int_{p}\left(Q_{\alpha \alpha }^{21}g_{\alpha \alpha }^{12}-Q_{\alpha \alpha }^{12}g_{\alpha \alpha }^{21}\right)\ln\frac{G_{\alpha \alpha }^{12}}{G_{\alpha \alpha }^{21}}\\ \sum_{\alpha }\int_{p,k}V_{\alpha }\left(k\right)\left[g_{\alpha \alpha }^{21}\left(p\right)G_{CC}^{12}\left(p+k\right)-g_{\alpha \alpha }^{12}\left(p\right)G_{CC}^{21}\left(p+k\right)\right]\ln\frac{g_{\alpha \alpha }^{21}\left(p\right)G_{CC}^{12}\left(p+k\right)}{g_{\alpha \alpha }^{12}\left(p\right)G_{CC}^{21}\left(p+k\right)}\geq 0, \end{array}$$

where we have used $\ln\frac{G_{\alpha \alpha }^{12}}{G_{\alpha \alpha }^{21}}=\ln\frac{f_{\alpha \alpha }}{1+f_{\alpha \alpha }}∼\ln\frac{g_{\alpha \alpha }^{12}}{g_{\alpha \alpha }^{21}}$ with Equations (90) and (91) and omitted *X* in the Green functions. We find that the tunneling of charged bosons contributes to the entropy production.

In a similar way, we can derive a kinetic entropy current for photons. Let us use the following relations for Fourier-transformed Green functions and self-energy for photons

(100) $$\begin{array}{c} D_{ij}^{ab}\left(X,p\right)=\left(\delta_{ij}-\frac{p_{i}p_{j}}{p^{2}}\right)D_{T}^{ab}\left(X,p\right)+\frac{p_{i}p_{j}}{p^{2}}D_{L}^{ab}\left(X,p\right), \end{array}$$

(101) $$\begin{array}{c} \Pi_{ij}^{ab}\left(X,p\right)=\left(\delta_{ij}-\frac{p_{i}p_{j}}{p^{2}}\right)\Pi_{T}^{ab}\left(X,p\right)+\frac{p_{i}p_{j}}{p^{2}}\Pi_{L}^{ab}\left(X,p\right), \end{array}$$

where $i\Pi =\frac{\delta \Gamma_{2}}{\delta D}$. Using the Kadanoff–Baym Ansatz $D_{CC,T}^{12}=-i\rho_{CC,T}f_{CC,T}$, $D_{CC,T}^{21}=-i\rho_{CC,T}\left(1+f_{CC,T}\right)$, $D_{CC,L}^{12}=-i\rho_{CC,L}f_{CC,L}$, $D_{CC,L}^{21}=-i\rho_{CC,L}\left(1+f_{CC,L}\right)$, $D_{\alpha \alpha ,T}^{12}=-i\rho_{\alpha \alpha ,T}f_{\alpha \alpha ,T}$, $D_{\alpha \alpha ,T}^{21}=-i\rho_{\alpha \alpha ,T}\left(1+f_{\alpha \alpha ,T}\right)$, $D_{\alpha \alpha ,L}^{12}=-i\rho_{\alpha \alpha ,L}f_{\alpha \alpha ,L}$, and $D_{\alpha \alpha ,L}^{21}=-i\rho_{\alpha \alpha ,L}\left(1+f_{\alpha \alpha ,L}\right)$ with $\rho_{CC,T}\equiv i\left(D_{CC,T}^{21}-D_{CC,T}^{12}\right)$, $\rho_{CC,L}\equiv i\left(D_{CC,L}^{21}-D_{CC,L}^{12}\right)$, $\rho_{\alpha \alpha ,T}\equiv i\left(D_{\alpha \alpha ,T}^{21}-D_{\alpha \alpha ,T}^{12}\right)$, $\rho_{\alpha \alpha ,L}\equiv i\left(D_{\alpha \alpha ,L}^{21}-D_{\alpha \alpha ,L}^{12}\right)$, $d_{\alpha \alpha ,\rho ,T}\equiv i\left(d_{\alpha \alpha ,T}^{21}-d_{\alpha \alpha ,T}^{12}\right)$, $d_{\alpha \alpha ,\rho ,L}\equiv i\left(d_{\alpha \alpha ,L}^{21}-d_{\alpha \alpha ,L}^{12}\right)$, $\Pi_{CC,\rho ,T}\equiv i\left(\Pi_{CC,T}^{21}-\Pi_{CC,T}^{12}\right)$, $\Pi_{CC,\rho ,L}\equiv i\left(\Pi_{CC,L}^{21}-\Pi_{CC,L}^{12}\right)$, $\Pi_{\alpha \alpha ,\rho ,T}\equiv i\left(\Pi_{\alpha \alpha ,T}^{21}-\Pi_{\alpha \alpha ,T}^{12}\right)$, $\Pi_{\alpha \alpha ,\rho ,L}\equiv i\left(\Pi_{\alpha \alpha ,L}^{21}-\Pi_{\alpha \alpha ,L}^{12}\right)$, and neglecting the 2nd order in the gradient expansion, we arrive at

(102) $$\begin{array}{cc} \partial_{\mu }s_{\mathrm{photon}}^{\mu }= & \frac{1}{2}\left(d-1\right)\int_{p}\left(\Pi_{CC,T}^{12}D_{CC,T}^{21}-\Pi_{CC,T}^{21}D_{CC,T}^{12}\right)\ln\frac{D_{CC,T}^{12}}{D_{CC,T}^{21}}\\ & +\frac{1}{2}\int_{p}\left(\Pi_{CC,L}^{12}D_{CC,L}^{21}-\Pi_{CC,L}^{21}D_{CC,L}^{12}\right)\ln\frac{D_{CC,L}^{12}}{D_{CC,L}^{21}}\\ & +\sum_{\alpha }\left(CC\to \alpha \alpha \right)\\ & +(\mathrm{tunneling}\;\mathrm{of}\;\mathrm{photons}), \end{array}$$

with

(103) $$\begin{array}{cc} \left(\mathrm{tunneling}\;\mathrm{of}\;\mathrm{photons}\right) & =\frac{1}{2}\sum_{\alpha }v_{a,\alpha }^{2}[\left(d-1\right)\int_{p}\left(d_{\alpha \alpha ,T}^{21}D_{CC,T}^{12}-d_{\alpha \alpha ,T}^{12}D_{CC,T}^{21}\right)\ln\frac{d_{\alpha \alpha ,T}^{21}D_{CC,T}^{12}}{d_{\alpha \alpha ,T}^{12}D_{CC,T}^{21}}\\ & \;+\int_{p}\left(d_{\alpha \alpha ,L}^{21}D_{CC,L}^{12}-d_{\alpha \alpha ,L}^{12}D_{CC,L}^{21}\right)\ln\frac{d_{\alpha \alpha ,L}^{21}D_{CC,L}^{12}}{d_{\alpha \alpha ,L}^{12}D_{CC,L}^{21}}]\geq 0, \end{array}$$

where $s_{\mathrm{photon}}^{\mu }=s_{\mathrm{photon},C}^{\mu }+\sum_{\alpha }s_{\mathrm{photon},\alpha }^{\mu }$ with

(104) sphoton,Cμ=∫p[(d−1)[pμ−12∂ReΠCC,R,T−∑αva,α2dαα,R,T∂pμρCC,Ti +12∂ReDCC,R,T∂pμΠCC,ρ,T−∑αva,α2dαα,ρ,Ti]σ[fCC,T] +[pμ−12∂ReΠCC,R,L−∑αva,α2dαα,R,L∂pμρCC,Li +12∂ReDCC,R,L∂pμΠCC,ρ,L−∑αva,α2dαα,ρ,Li]σ[fCC,L]],sphoton,αμ=∫p[(d−1)[pμ−12∂ReΠαα,R,T∂pμραα,Ti+12∂ReDαα,R,T∂pμΠαα,ρ,Ti

(105) +12va,α2∂ReDCC,R,T∂pμdαα,ρ,Ti−∂Redαα,R,T∂pμρCC,Ti]σ[fαα,T]+[pμ−12∂ReΠαα,R,L∂pμραα,Li+12∂ReDαα,R,L∂pμΠαα,ρ,Li

(106) +12va,α2∂ReDCC,R,L∂pμdαα,ρ,Li−∂Redαα,R,L∂pμρCC,Li]σ[fαα,L]].

We now show that the sum of the 1st term on the right-hand side in Equation (95) and the 1st and the 2nd term on the right-hand side in Equation (102) is semi-positive definite for $O(e^{2})$ and $O(e^{4}|\bar{\varphi }{|}^{2})$ self-energy (the Hartree–Fock approximation). We also show that the sum of the 2nd term on the right-hand side in Equation (95) and the 3rd term on the right-hand-side in Equation (102) is semi-positive definite for the $O(e^{2})$ and $O(e^{4}|\bar{\varphi }{|}^{2})$ self-energy. The proof is the same as that in the isolated system in [58].

Hence, we find that

(107) $$\begin{array}{c} \partial_{\mu }s^{\mu }\geq 0, \end{array}$$

for $s^{\mu }=s_{\mathrm{matter}}^{\mu }+s_{\mathrm{photon}}^{\mu }$ in the LO of the tunneling variable and coupling expansion in the Hartree–Fock approximation in the 1st order in the gradient expansion. For the equilibrium state, we arrive at

(108) $$\begin{array}{ccc} f_{CC,T\mathrm{or}L}\left(p\right) & = & f_{\alpha \alpha ,T\mathrm{or}L}\left(p\right)=\frac{1}{\exp\left(\frac{p^{0}}{T}\right)-1},\\ f_{CC}\left(p\right) & = & \frac{1}{\exp\left(\frac{p^{0}-\mu_{C}}{T}\right)-1},\;\;\;\;f_{\alpha \alpha }\left(p\right)=\frac{1}{\exp\left(\frac{p^{0}-\mu_{\alpha }}{T}\right)-1},\\ \mu_{C} & = & -e\left(A_{C}^{0}-\frac{\partial^{0}\beta_{C}}{e}\right),\;\;\;\;\mu_{\alpha }=-e\left(A_{\alpha }^{0}-\frac{\partial^{0}\beta_{\alpha }}{e}\right),\\ \mu_{C} & = & \mu_{\alpha }, \end{array}$$

where *T* is absolute temperature, $\mu_{C}$ and $\mu_{\alpha }$ are the chemical potentials for *C* and α=L,R, respectively. Chemical potentials are negative signs of gauge invariant parts of scalar potentials. This is derived in the proof of the H-theorem for the $O(e^{4}|\bar{\varphi }{|}^{2})$ self-energy in the Appendix A. Due to the tunneling processes in Equation (99), temperature and the chemical potential in *C* and α=L,R are the same values in the equilibrium state.

## 5. Time Evolution Equations in Spatially-Homogeneous Systems in Open Systems

In this section we derive the Klein–Gordon (KG) equations for coherent fields and the Kadanoff–Baym (KB) equations for quantum fluctuations in the spatially-homogeneous system. In this section, we use the α=L,R to represent the two reservoirs; the *R* represents the ’retarded’ and *L* represents the ’longitudinal’ reservoir of photons.

We introduce the statistical functions $F_{CC}\equiv \frac{G_{CC}^{21}+G_{CC}^{12}}{2},$ $F_{\alpha \alpha }\equiv \frac{G_{\alpha \alpha }^{21}+G_{\alpha \alpha }^{12}}{2},$ $g_{\alpha \alpha ,F}\equiv \frac{g_{\alpha \alpha }^{21}+g_{\alpha \alpha }^{12}}{2},$ $F_{CC,T}\equiv \frac{D_{CC,T}^{21}+D_{CC,T}^{12}}{2},$ $F_{CC,L}\equiv \frac{D_{CC,L}^{21}+D_{CC,L}^{12}}{2},$ $F_{\alpha \alpha ,T}\equiv \frac{D_{\alpha \alpha ,T}^{21}+D_{\alpha \alpha ,T}^{12}}{2},$ $F_{\alpha \alpha ,L}\equiv \frac{D_{\alpha \alpha ,L}^{21}+D_{\alpha \alpha ,L}^{12}}{2},$ $d_{\alpha \alpha ,F,T}\equiv \frac{d_{\alpha \alpha ,T}^{21}+d_{\alpha \alpha ,T}^{12}}{2},$ $d_{\alpha \alpha ,F,L}\equiv \frac{d_{\alpha \alpha ,L}^{21}+d_{\alpha \alpha ,L}^{12}}{2},$ in addition to the spectral functions $\rho_{CC}\equiv i\left(G_{CC}^{21}-G_{CC}^{12}\right)$, $\rho_{\alpha \alpha }\equiv i\left(G_{\alpha \alpha }^{21}-G_{\alpha \alpha }^{12}\right)$, $g_{\alpha \alpha ,\rho }\equiv i\left(g_{\alpha \alpha }^{21}-g_{\alpha \alpha }^{12}\right)$, $\rho_{CC,T}\equiv i\left(D_{CC,T}^{21}-D_{CC,T}^{12}\right)$, $\rho_{CC,L}\equiv i\left(D_{CC,L}^{21}-D_{CC,L}^{12}\right)$, $\rho_{\alpha \alpha ,T}\equiv i\left(D_{\alpha \alpha ,T}^{21}-D_{\alpha \alpha ,T}^{12}\right)$, $\rho_{\alpha \alpha ,L}\equiv i\left(D_{\alpha \alpha ,L}^{21}-D_{\alpha \alpha ,L}^{12}\right)$. $d_{\alpha \alpha ,\rho ,T}\equiv i\left(d_{\alpha \alpha ,T}^{21}-d_{\alpha \alpha ,T}^{12}\right)$, We also introduce the following two types of self-energy, $\Sigma_{CC,F}\equiv \frac{\Sigma_{CC}^{21}+\Sigma_{CC}^{12}}{2},\Sigma_{\alpha \alpha ,F}\equiv \frac{\Sigma_{\alpha \alpha }^{21}+\Sigma_{\alpha \alpha }^{12}}{2},\Pi_{CC,F,T}\equiv \frac{\Pi_{CC,T}^{21}+\Pi_{CC,T}^{12}}{2},\Pi_{CC,F,L}\equiv \frac{\Pi_{CC,L}^{21}+\Pi_{CC,L}^{12}}{2},\Pi_{\alpha \alpha ,F,T}\equiv \frac{\Pi_{\alpha \alpha ,T}^{21}+\Pi_{\alpha \alpha ,T}^{12}}{2},\Pi_{\alpha \alpha ,F,L}\equiv \frac{\Pi_{\alpha \alpha ,L}^{21}+\Pi_{\alpha \alpha ,L}^{12}}{2},$ $\Sigma_{CC,\rho }\equiv i\left(\Sigma_{CC}^{21}-\Sigma_{CC}^{12}\right),$ $\Sigma_{\alpha \alpha ,\rho }\equiv i\left(\Sigma_{\alpha \alpha }^{21}-\Sigma_{\alpha \alpha }^{12}\right),$ $\Pi_{CC,\rho ,T}\equiv i\left(\Pi_{CC,T}^{21}-\Pi_{CC,T}^{12}\right),$ $\Pi_{CC,\rho ,L}\equiv i\left(\Pi_{CC,L}^{21}-\Pi_{CC,L}^{12}\right),$ $\Pi_{\alpha \alpha ,\rho ,L}\equiv i\left(\Pi_{\alpha \alpha ,T}^{21}-\Pi_{\alpha \alpha ,T}^{12}\right),$ and $\Pi_{\alpha \alpha ,\rho ,L}\equiv i\left(\Pi_{\alpha \alpha ,L}^{21}-\Pi_{\alpha \alpha ,L}^{12}\right).$ We then derive the following Kadanoff–Baym equations from Equations (48) and (53):

(109) p2−m2−ΣCC,loc−ReΣCC,R+∑αReUαα,R,FCCC+ReGCC,R,ΣCC,F−∑αUαα,FC=1iFCCΣCC,ρ−ρCCΣCC,F−1i∑αFCCUαα,ρ−ρCCUαα,F,

(110) p2−m2−ΣCC,loc−ReΣCC,R+∑αReUαα,R,ρCCC+ReGCC,R,ΣCC,ρ−∑αUαα,ρC=0,

where $U_{\alpha \alpha ,F}\equiv \frac{U_{\alpha \alpha }^{21}+U_{\alpha \alpha }^{12}}{2}$ and $U_{\alpha \alpha ,\rho }\equiv i\left(U_{\alpha \alpha }^{21}-U_{\alpha \alpha }^{12}\right)$ with $U_{\alpha \alpha }^{12\;\mathrm{or}\;21}\left(X,p\right)=\int_{k}V_{\alpha }\left(X,k\right)g_{\alpha \alpha }^{12\;\mathrm{or}\;21}\left(X,p-k\right)$ and $V_{\alpha }\left(X,k\right)={\left|v_{\alpha }\right|}^{2}{\left(2\pi \right)}^{d+1}\delta^{d+1}\left(k+e\left(A_{C}-\frac{\partial \beta_{C}}{e}\right)-e\left(A_{\alpha }-\frac{\partial \beta_{\alpha }}{e}\right)-\partial \theta_{\alpha }\right)$ as given in Equation (98). We derive the following equations from Equations (57) and (58):

(111) p2−m2−Σαα,loc−ReΣαα,R,gαα,Fα+Regαα,R,Σαα,Fα=1igαα,FΣαα,ρ−gαα,ρΣαα,F,

(112) p2−m2−Σαα,loc−ReΣαα,R,gαα,ρα+Regαα,R,Σαα,ρα=0.

We obtain the following equations from Equations (68) and (69):

(113) p2−m2−Σαα,loc−ReΣαα,R,Fααα+ReGαα,R,Σαα,Fα+ReQαα,R,gαα,Fα−Regαα,R,Qαα,Fα=1iFααΣαα,ρ−ρααΣαα,F−1igαα,FQαα,ρ−gαα,ρQαα,F,

(114) p2−m2−Σαα,loc−ReΣαα,R,ρααα+ReGαα,R,Σαα,ρα+ReQαα,R,gαα,ρα−Regαα,R,Qαα,ρα=0,

where $Q_{\alpha \alpha ,F}\equiv \frac{Q_{\alpha \alpha }^{21}+Q_{\alpha \alpha }^{12}}{2}$ and $Q_{\alpha \alpha ,\rho }\equiv i\left(Q_{\alpha \alpha }^{21}-Q_{\alpha \alpha }^{12}\right)$ with $Q_{\alpha \alpha }^{12\;\mathrm{or}\;21}\left(X,p\right)=\int_{k}V_{\alpha }\left(X,k\right)G_{CC}^{12\;\mathrm{or}\;21}\left(X,p+k\right)$.

Similarly, we derive the Kadanoff–Baym equations for photons as

(115) p2−2e2|φ¯C|2−ΠCC,loc,T−ReΠCC,R,T+Re∑αva,α2dαα,R,FCC,T+ReDCC,R,T,ΠCC,F,T−∑αva,α2dαα,F=1i(FCC,TΠCC,ρ,T−ρCC,TΠCC,F,T)−1i∑αva,α2(FCC,Tdαα,ρ,T−ρCC,Tdαα,F,T),

(116) p2−2e2|φ¯C|2−ΠCC,loc,T−ReΠCC,R,T+Re∑αva,α2dαα,R,ρCC,T+ReDCC,R,T,ΠCC,ρ,T−∑αva,α2dαα,ρ=0,

(117) p2−2e2|φ¯α|2−Παα,loc,T−ReΠαα,R,T,dαα,F,T+Redαα,R,T,Παα,F,T=1i(dαα,F,TΠαα,ρ,T−dαα,ρ,TΠαα,F,T),

(118) p2−2e2|φ¯α|2−Παα,loc,T−ReΠαα,R,T,dαα,ρ,T+Redαα,R,T,Παα,ρ,T=0,

(119) p2−2e2|φ¯α|2−Παα,loc,T−ReΠαα,R,T,Fαα,T+ReDαα,R,T,Παα,F,T+va,α2ReDCC,R,T,dαα,F,T−Redαα,R,T,va,α2FCC,T=1i(Fαα,TΠαα,ρ,T−ραα,TΠαα,F,T)−1iva,α2(dαα,F,TρCC,T−dαα,ρ,TFCC,T),

(120) p2−2e2|φ¯α|2−Παα,loc,T−ReΠαα,R,T,ραα,T+ReDαα,R,T,Παα,ρ,T+va,α2ReDCC,R,T,dαα,ρ,T−Redαα,R,T,va,α2ρCC,T=0.

The Kadanoff–Baym equations for longitudinal modes are given by changing the label *T* to *L* in the above equations.

Next, we write the Klein–Gordon equations for coherent fields. We use ${\bar{\varphi }}_{C}=\left|{\bar{\varphi }}_{C}\right|e^{i\beta_{C}}$, ${\bar{\varphi }}_{\alpha }=\left|{\bar{\varphi }}_{\alpha }\right|e^{i\beta_{\alpha }}$ with α=L,R, and $v_{\alpha }=\left|v_{\alpha }\right|e^{i(\beta_{C}-\beta_{\alpha }+\theta_{\alpha })}$. Multiplying $e^{-i\beta_{C}\left(X\right)}$ in Equation (25) and taking the real part, we arrive at

(121) ∂02|φ¯C|=e2AC0−∂0βCe2|φ¯C|−e2ACi−∂iβCe2|φ¯C| −m2+(d−1)e2∫pFCC,T(X,p)+e2∫pFCC,L(X,p)−(counterterms)|φ¯C| +2e4|φ¯C|∫p[ReGCC,RX,p−e(AC−∂βC/e)PCC,F(X,p) +FCC(X,p−e(AC−∂βC/e))RePCC,R(X,p)] +∑α|vα||φ¯α|cosθα(X),

(122) $$\begin{array}{ccc} \left(\mathrm{counter}\;\mathrm{terms}\right) & = & de^{2}\int \frac{d^{d}p}{{\left(2\pi \right)}^{d}}\frac{1}{2\sqrt{p^{2}+2e^{2}{\left|{\bar{\varphi }}_{C}\right|}^{2}}}, \end{array}$$

where $P_{CC,F}\left(X,p\right)$ and $P_{CC,R}\left(X,p\right)$ are the Fourier transformations of $P_{CC,F}\left(x,y\right)\equiv \frac{P^{21}\left(x,y\right)+P^{12}\left(x,y\right)}{2}$ and $P_{CC,R}\left(x,y\right)\equiv i\left(P_{CC}^{11}\left(x,y\right)-P_{CC}^{12}\left(x,y\right)\right)$ with $P_{CC}^{ab}\left(x,y\right)=D_{CC,ij}^{ab}\left(x,y\right)D_{CC,ij}^{ab}\left(x,y\right)$. Here, we have left the 0th order terms in the gradient expansion on the right-hand side. We then write the following equations by using Equation (27):

(123) ∂02|φ¯α|=e2Aα0−∂0βαe2|φ¯α|−e2Aαi−∂iβαe2|φ¯α| −m2+(d−1)e2∫pFαα,T(X,p)+e2∫pFαα,L(X,p)−(counterterms)|φ¯α| +2e4|φ¯α|∫p[ReGαα,RX,p−e(Aα−∂βα/e)Pαα,F(X,p) +Gαα,F(X,p−e(Aα−∂βα/e))RePαα,R(X,p)] +|vα||φ¯C|cosθα(X),

(124) $$\begin{array}{ccc} \left(\mathrm{counter}\;\mathrm{terms}\right) & = & de^{2}\int \frac{d^{d}p}{{\left(2\pi \right)}^{d}}\frac{1}{2\sqrt{p^{2}+2e^{2}{\left|{\bar{\varphi }}_{\alpha }\right|}^{2}}}, \end{array}$$

where $P_{\alpha \alpha }^{ab}\left(X,p\right)$ is the Fourier transformation of $P_{\alpha \alpha }^{ab}\left(x,y\right)=D_{\alpha \alpha ,ij}^{ab}\left(x,y\right)D_{\alpha \alpha ,ij}^{ab}\left(x,y\right)$.

Let us multiply $i{\bar{\varphi }}_{C}^{*}$ in Equation (25) and $i{\bar{\varphi }}_{C}$ in Equation (26), and take the difference, then we arrive at

(125) $$\begin{array}{cc} \partial^{0}i\left[-{\bar{\varphi }}_{C}^{*}\left(\partial_{0}-ieA_{C,0}\right){\bar{\varphi }}_{C}+\left(\left(\partial_{0}+ieA_{C,0}\right){\bar{\varphi }}_{C}^{*}\right){\bar{\varphi }}_{C}\right] & \\ +i\sum_{\alpha }\left(v_{\alpha }{\bar{\varphi }}_{C}^{*}{\bar{\varphi }}_{\alpha }-v_{\alpha }^{*}{\bar{\varphi }}_{C}{\bar{\varphi }}_{\alpha }^{*}\right)+\frac{i}{2}\left[{\bar{\varphi }}_{C}^{*}\frac{\delta \Gamma_{2}}{\delta {\bar{\varphi }}_{C}^{*}}-{\bar{\varphi }}_{C}\frac{\delta \Gamma_{2}}{\delta {\bar{\varphi }}_{C}}\right] & =0. \end{array}$$

For the $O(e^{2})$ and $O(e^{4}|\bar{\varphi }{|}^{2})$ diagrams (the Hartree–Fock approximation), we can use

(126) i2φ¯C*δΓ2δφ¯C*−φ¯CδΓ2δφ¯C=−∂0∫pReΣCC,R∂FCC∂p0+ΣCC,F∂ReGCC,R∂p0 +1i∫pFCCΣCC,ρ−ρCCΣCC,F=∂0∫p2p0FCC−∂0∑α∫pReUαα,R∂FCC∂p0+Uαα,F∂ReGCC,R∂p0 +1i∫pFCCUαα,ρ−ρCCUαα,F,

where we have used the relation in the Appendix A and the integration ($\int_{p}$) of the KB equation (109). Then we can write Equation (125) as

(127) −∂02|φ¯C|2eAC0−∂0βCe+∂0∫p2p0FCC=+2∑α|vα||φ¯C||φ¯α|sinθα+∂0∑α∫pReUαα,R∂FCC∂p0+Uαα,F∂ReGCC,R∂p0−1i∑α∫pFCCUαα,ρ−ρCCUαα,F.

The left-hand side represents the time derivative of the charge in the *C*. This is equivalent to the tunneling of charged bosons between the *C* and the α=L,R reservoirs on the right-hand side. The first term on the right-hand side represents the Josephson current. We interpret the above equation as representing the time evolution equation for $A_{C}^{0}-\frac{\partial^{0}\beta_{C}}{e}$. Similarly, we derive

(128) −∂02|φ¯α|2eAα0−∂0βαe+∂0∫p2p0Fαα=−2|vα||φ¯C||φ¯α|sinθα+∂0∫pReQαα,R∂gαα,F∂p0+Qαα,F∂Regαα,R∂p0−1i∫pgαα,FQαα,ρ−gαα,ρQαα,F,

where we have used the integration of Equation (113). We now show the total charge conservation as

(129) $$\begin{array}{ccc} -\partial_{0}\left[2|{\bar{\varphi }}_{C}{|}^{2}e\left(A_{C}^{0}-\frac{\partial^{0}\beta_{C}}{e}\right)\right]+\partial_{0}\int_{p}2p^{0}F_{CC} & \\ -\partial_{0}\sum_{\alpha }\left[2|{\bar{\varphi }}_{\alpha }{|}^{2}e\left(A_{\alpha }^{0}-\frac{\partial^{0}\beta_{\alpha }}{e}\right)\right]+\sum_{\alpha }\partial_{0}\int_{p}2p^{0}F_{\alpha \alpha } & = & 0, \end{array}$$

by using the definitions $U_{\alpha \alpha }\left(X,p\right)=\int_{k}V_{\alpha }\left(X,k\right)g_{\alpha \alpha }\left(X,p-k\right)$ and $Q_{\alpha \alpha }\left(X,p\right)=\int_{k}V_{\alpha }\left(X,k\right)G_{CC}\left(X,p+k\right)$. We set the total charge to be zero in the spatially-homogeneous system. Using Equations (21) and (22), the Klein–Gordon equations for $A_{C}^{i}$ and $A_{\alpha }^{i}$ are written as

(130) $$\begin{array}{ccc} \partial_{0}^{2}\left(A_{C}^{i}-\frac{\partial^{i}\beta_{C}}{e}\right) & = & -2e^{2}{\left|{\bar{\varphi }}_{C}\right|}^{2}\left(A_{C}^{i}-\frac{\partial^{i}\beta_{C}}{e}\right)+2e\int_{p}p^{i}F_{CC}-\frac{1}{2}\frac{\delta \Gamma_{2}}{\delta A_{C,i}}, \end{array}$$

(131) $$\begin{array}{ccc} \partial_{0}^{2}\left(A_{\alpha }^{i}-\frac{\partial^{i}\beta_{\alpha }}{e}\right) & = & -2e^{2}{\left|{\bar{\varphi }}_{\alpha }\right|}^{2}\left(A_{\alpha }^{i}-\frac{\partial^{i}\beta_{\alpha }}{e}\right)+2e\int_{p}p^{i}F_{\alpha \alpha }-\frac{1}{2}\frac{\delta \Gamma_{2}}{\delta A_{\alpha ,i}}, \end{array}$$

When $F_{CC}$ and $F_{\alpha \alpha }$ is symmetric under $p^{i}\to -p^{i}$ at the initial time, we find the solutions $A_{C}^{i}-\frac{\partial^{i}\beta_{C}}{e}=A_{\alpha }^{i}-\frac{\partial^{i}\beta_{\alpha }}{e}=0$ at any point in time, which are derived in the same way as the isolated system [58].

We find that Equation (99) represents the entropy production in tunneling processes between *C* and α reservoirs. Here, we discuss only the tunneling phenomena which never change the frequency. Then, we just impose the following constraint on parameters $\theta_{\alpha }$:

(132) $$\begin{array}{c} \partial^{0}\theta_{\alpha }=e\left(A_{C}^{0}-\frac{\partial^{0}\beta_{C}}{e}\right)-e\left(A_{\alpha }^{0}-\frac{\partial^{0}\beta_{\alpha }}{e}\right). \end{array}$$

Finally, we write the total conserved energy. Using the KB equations and the KG equations and assuming the $|v_{\alpha }|$’s are constants, we derive the total energy as

(133) $$\begin{array}{ccc} E_{\mathrm{tot}} & = & E_{\mathrm{coh}}+E_{\mathrm{qf}}+E_{\mathrm{pot},\mathrm{loc}}+E_{\mathrm{pot},\mathrm{nonl}}, \end{array}$$

(134) $$\begin{array}{cc} E_{\mathrm{coh}} & ={\left(\partial_{0}\left|{\bar{\varphi }}_{C}\right|\right)}^{2}+\frac{1}{2}{\left(\partial_{0}\left(A_{C}^{i}-\frac{\partial^{i}\beta_{C}}{e}\right)\right)}^{2}\\ & +m^{2}|{\bar{\varphi }}_{C}{|}^{2}+e^{2}{\left(A_{C}^{0}-\frac{\partial^{0}\beta_{C}}{e}\right)}^{2}|{\bar{\varphi }}_{C}{|}^{2}+e^{2}{\left(A_{C}^{i}-\frac{\partial^{i}\beta_{C}}{e}\right)}^{2}{\left|{\bar{\varphi }}_{C}\right|}^{2}\\ & +\sum_{\alpha }\left(C\to \alpha \right)\\ & -\sum_{\alpha }2|v_{\alpha }\left|\right|{\bar{\varphi }}_{C}\left|\right|{\bar{\varphi }}_{\alpha }|\cos\theta_{\alpha }, \end{array}$$

(135) $$\begin{array}{cc} E_{\mathrm{qf}} & =\int_{p}2{\left(p^{0}\right)}^{2}F_{CC}\left(X,p\right)+\frac{1}{2}\int_{p}2{\left(p^{0}\right)}^{2}\left(\left(d-1\right)F_{CC,T}\left(X,p\right)+F_{CC,L}\left(X,p\right)\right)\\ & +\sum_{\alpha }\left(CC\to \alpha \alpha \right), \end{array}$$

(136) $$\begin{array}{cc} E_{\mathrm{pot},\mathrm{loc}} & =-e^{2}\int_{k}\left[\left(d-1\right)F_{CC,T}\left(X,k\right)+F_{CC,L}\left(X,k\right)\right]\int_{l}F_{CC}\left(X,l\right)\\ & +\left(\left(d-1\right)\delta m_{C,T}^{2}+\delta m_{C,L}^{2}\right)\int_{l}F_{CC}\left(X,l\right)\\ & +\frac{1}{2}\delta m_{\mathrm{cb}}^{2}\int_{k}\left[\left(d-1\right)F_{CC,T}\left(X,k\right)+F_{CC,L}\left(X,k\right)\right]\\ & +\sum_{\alpha }\left(CC\to \alpha \alpha \right), \end{array}$$

(137) Epot,nonl=−13∫pReΣCC,R(X,p)FCC(X,p)+ΣCC,F(X,p)ReGCC,R(X,p)−d−16∫pReΠCC,R,T(X,p)FCC,T(X,p)+ΠCC,F,T(X,p)ReDCC,R,T(X,p)−16∫pReΠCC,R,L(X,p)FCC,L(X,p)+ΠCC,F,L(X,p)ReDCC,R,L(X,p)+∑αCC→αα,

where $\delta m_{cb}^{2}=2e^{2}\int_{p}\frac{1}{2\sqrt{p^{2}+m^{2}}}$, $\delta m_{C,T\mathrm{or}L}^{2}=e^{2}\int_{p}\frac{1}{2\sqrt{p^{2}+2e^{2}{\left|{\bar{\varphi }}_{C}\right|}^{2}}}$, and $\delta m_{\alpha ,T\mathrm{or}L}^{2}=e^{2}\int_{p}\frac{1}{2\sqrt{p^{2}+2e^{2}{\left|{\bar{\varphi }}_{\alpha }\right|}^{2}}}$. The notations $\left(C\to \alpha \right)$ and $\left(CC\to \alpha \alpha \right)$ represent the terms changing the labeling *C* to α for coherent fields and the labeling CC to αα in the Green functions and the self-energy in the previous terms, respectively.

## 6. Discussion

In this paper, we have derived the Klein–Gordon (KG) equations and the Kadanoff–Baym (KB) equations to describe non-equilibrium phenomena in quantum electrodynamics (QED) with charged bosons in open systems. We have found that time evolution equations of diagonal elements in KB equations are written by gauge-invariant Green functions and self-energy for the Type I gauge transformation to the 1st order in the gradient expansion. We have introduced a kinetic entropy current in QED for open systems to the 1st order approximation in the gradient expansion, and shown the H-theorem in the Hartree–Fock approximation and in LO of the tunneling variable expansion. We have shown that the tunneling processes also contribute to entropy production. We have written the KG equations and the KB equations only with real and pure imaginary functions in the spatially-homogeneous system, namely real statistical functions, pure imaginary spectral functions, and absolute values of coherent fields of charged bosons. It is possible to show the charge-energy conservation in the total system, and no memory integral terms appear in the conserved energy in the Hartree–Fock approximation, in 1st order approximation in the gradient expansion.

It is important to discuss equilibrium states. In an equilibrium state, the central region *C* and the α=L,R reservoirs have the same temperature and chemical potentials due to the tunneling processes as shown in the proof of the H-theorem. The chemical potentials are negative signs of gauge-invariant parts of scalar potentials as shown in the Appendix A. When scalar potentials have the same constant values, the $\partial^{0}\theta_{\alpha }$ in the time derivative of phase factors in the tunneling variables in Equation (132) is zero, namely $\theta_{\alpha }=\mathrm{constant}$. Since the tunneling processes are balanced in the central region *C* and the α=L,R reservoirs and charge flow between systems stops, we find $\theta_{\alpha }=0$ or π due to Equations (127) and (128). The ratios of the coherent fields of charged bosons $|{\bar{\varphi }}_{C}|$ and $|{\bar{\varphi }}_{\alpha }|$ are determined by setting the left-hand side in Equations (121) and (123) to zero with the same scalar potentials. Since the proof of the H-theorem restricts the distribution functions but does not restrict the dispersion relations for equilibrium states, the $|{\bar{\varphi }}_{C}|$ and the $|{\bar{\varphi }}_{\alpha }|$ (the mass of evanescent photons) might have different values. When the 2nd derivatives on the left-hand side in Equations (121) and (123) remains, the coherent fields of charged bosons $|{\bar{\varphi }}_{C}|$ and $|{\bar{\varphi }}_{\alpha }|$ might oscillate around the minimum value of the potential energy $\Phi (|\bar{\varphi }\left|\right)=m^{2}|\bar{\varphi }{|}^{2}+e^{2}{\left(A^{0}-\frac{\partial^{0}\beta }{e}\right)}^{2}|\bar{\varphi }{|}^{2}=m^{2}{\left|\bar{\varphi }\right|}^{2}+\frac{c_{1}}{|\bar{\varphi }{|}^{2}}$ in the 3rd and the 4th terms in Equation (134) in $\left(A^{0}-\frac{\partial^{0}\beta }{e}\right)=\frac{c_{1}}{e|\bar{\varphi }{|}^{2}}$ with $c_{1}$ proportional to the total charge of incoherent particles in the system as in the case of the isolated system [58]. Since the mass of evanescent photons oscillates due to the 2nd order contributions in the gradient expansion, Green functions still oscillate at later times near the equilibrium state.

**Figure 3:**
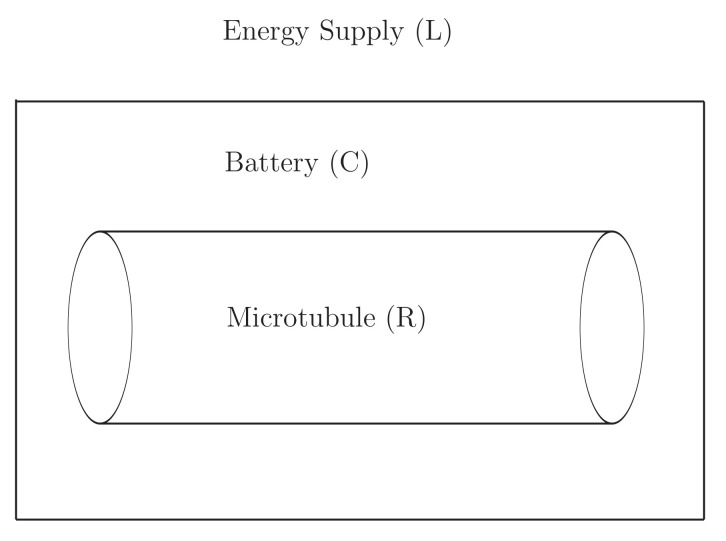
Open systems rewritten by energy supply (L), battery (C), and microtubule laser (R).

We have considered three regions as a practical example of an open system. They are rewritten by energy supply (L), water battery (C), and microtubule (R) shown in Figure 3. The energy supply provides incoherent photons to achieve coherent output of water dipoles and photons inside microtubules. The source of photons, which might be mitochondria or reactive-oxygen species (ROS) in living cells [73], is still largely unknown. The water battery plays the role of maintaining coherent states inside microtubules. It will be interesting to investigate the relevant time scales required to maintain coherent states only by the use of the water battery in case no external energy being supplied. We might be able to describe the formation of coherent states in these systems.

We also discuss the equilibration processes in the central region and multiple reservoirs $\alpha =1,2,\cdots ,N_{\mathrm{res}}$, that is, the network. In QBD, there are at least two types of quantum mechanisms for information transfer between systems. The first one is to use self-induced transparency in microtubules which connect two coherent regions [30]. Pulse propagation appears from one side of a microtubule to the opposite end, then the information transfer between coherent regions takes place. The second one is to use quantum tunneling phenomena [31]. In case several coherent regions are surrounded by non-coherent regions and the distances between two coherent regions are smaller than the inverse of mass of evanescent photons, the coherent field transfer and the incoherent particle transfer between systems can occur. We now describe the information transfer with quantum tunneling phenomena in this case. The second one is the same as the Josephson effect, which suggests the tunneling current between two superconducting regions separated by the normal metal regions. We have also shown that the Josephson current appears in Equations (127) and (128) in QED with charged bosons. It is possible to extend our theory to the case of the network by changing $\sum_{\alpha =L,R}$ to $\sum_{\alpha =1}^{N_{\mathrm{res}}}$ in all the time evolution equations. If we trace time evolution in the network, we might be able to describe not only the equilibration but also the information transfer among regions in the brain.

In this paper, although we have discussed time evolution equations with relativistic charged bosons, our analysis is applied to a non-relativistic case. In the non-relativistic case, we need to change from $iG_{0}^{-1}\left(p\right)=p^{2}-m^{2}$ to $iG_{0}^{-1}\left(p\right)=p^{0}-\frac{p^{2}}{2m}$ in the KB equations for charged bosons, and multiply by the factor $\frac{1}{4m^{2}}$ in all of the nonlocal self-energy in the relativistic case in this paper.

We also need to extend our theory (QED with charged boson fields) to electric dipole fields in order to describe water electric dipoles in open systems. Since we have derived time evolution equations for charged boson fields with the gauge invariant functions in open systems, we only need the theory for dipole fields to perform multi-energy-mode analysis with the KG and the KB equations. Although we can check whether coherent states in QED with charged bosons are robust or not qualitatively, we need to describe time evolution for dipole fields in 3+1 dimensions to estimate quantitative behaviors of water electric dipole fields and photon fields in the memory formation in the brain.

## 7. Conclusions

In this paper we have derived the Klein–Gordon equations and the Kadanoff–Baym equations in QED with charged singlet bosons in open systems. These equations are expressed only by gauge invariant quantities in the 1st order in the gradient expansion. They describe non-equilibrium, charge-energy conserving, and entropy-producing dynamics in the Hartree–Fock approximation with the LO approximation of tunneling variable and coupling expansion in the 1st order in the gradient expansion. This work paves the way for a concrete implementation of this approach to the modeling of quantum brain dynamics, which until now has not incorporated open system characteristics of the brain. Both metabolic energy supply and thermal dissipation need to be accounted for in addition to the nonlinear interactions between the quantum fields (Figure 3). The remaining challenge is to represent realistic dynamical degrees of freedom that correspond to information storage and information processing capabilities in neurons and neuronal assemblies. This will not only require a sufficiently long decoherence time for these quantum fields (on the order of 1 ms or more) but also the formulation of testable predictions for such a model. While we are not yet prepared to propose a specific implementation of such a representation, the current model is sufficiently generalised that it covers the essential features expected of a properly formulated quantum brain dynamics theory. At present, the most likely candidate structures for a biophysical representation of the presented model appear to be neuronal microtubules, especially in dendrites. However, we still need to identify specific microscopic degrees of freedom which can be involved in quantum coherence through nonlinear interactions similar to those taking place in laser action. This also requires inclusion of incoherent energy pumping. As is the case with lasers, due to these nonlinear interactions between dynamic degrees of freedom, the pumped energy is transformed into quantum condensed modes, which can overcome the decoherence effects of thermal motion at physiological temperature. This biophysical model development can, for example, involve the interactions between tryptophan residues in tubulin dimers as recently argued by Craddock et al. [74]. While quantum coherence simulated in this work only survives about 1 ps in a single dimer, this can be extended to much longer decoherence times using the nonequilibrium quantum field theory formalism for an entire microtubule including energy pumping which the Craddock et al., paper did not consider. 

## Figures and Tables

**Figure A1:**
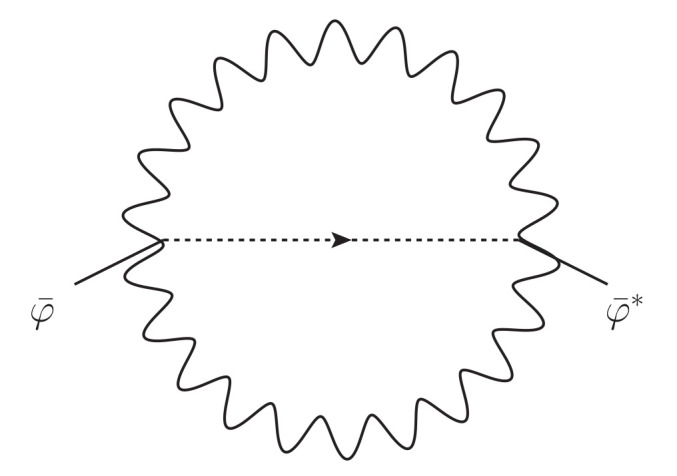
The $O(e^{4}|\bar{\varphi }{|}^{2})$ diagram in $\frac{\Gamma_{2}}{2}$.

## Appendix A. The *O*(*e*^4^|$\bar{\varphi }$|^2^) Diagram

In this section, we shall derive several relations with respect to the $O(e^{4}|\bar{\varphi }{|}^{2})$ diagram in the isolated system. We omit the subscript CC or αα with α=L,R.

The $\frac{i\Gamma_{2}^{\prime }}{2}$ in Figure A1 is

(A1) $$\begin{array}{c} \frac{i\Gamma_{2}^{\prime }}{2}=-2e^{4}\int_{C}d^{d+1}ud^{d+1}w\left[{\bar{\varphi }}^{*}\left(u\right)\bar{\varphi }\left(w\right)D^{\mu \nu }\left(u,w\right)D_{\nu \mu }\left(w,u\right)\Delta \left(w,u\right)\right]. \end{array}$$

The functional derivative by ${\bar{\varphi }}^{*}\left(x\right)$ is written as

(A2) $$\begin{array}{cc} \frac{\delta }{\delta {\bar{\varphi }}^{*}\left(x\right)}\frac{\Gamma_{2}^{\prime }}{2} & =\frac{-2e^{4}}{i}\int d^{d+1}w\bar{\varphi }\left(w\right)[D_{ij}^{11}\left(x,w\right)D_{ji}^{11}\left(w,x\right)\Delta^{11}\left(w,x\right)\\ & \;-D_{ij}^{12}\left(x,w\right)D_{ji}^{21}\left(w,x\right)\Delta^{21}\left(w,x\right)]\\ & =2e^{4}\int d^{d+1}w\left[P_{R}\left(x,w\right)\Delta_{F}\left(w,x\right)+P_{F}\left(x,w\right)\Delta_{A}\left(w,x\right)\right]\bar{\varphi }\left(w\right), \end{array}$$

with $P^{ab}\left(x,w\right)\equiv D_{ij}^{ab}\left(x,w\right)D_{ji}^{ba}\left(w,x\right)={\left(D_{ij}^{ab}\left(x,w\right)\right)}^{2}$ (a,b=1,2), $P_{R}\equiv i\left(P^{11}-P^{12}\right)$, $P_{F}\equiv \frac{P^{12}+P^{21}}{2}$, $\Delta_{R}\equiv i\left(\Delta^{11}-\Delta^{12}\right)$, $\Delta_{F}\equiv \frac{\Delta^{12}+\Delta^{21}}{2}$, and $\Delta_{A}=i\left(\Delta^{11}-\Delta^{21}\right)$. By multiplying $e^{-i\beta (x)}$ and taking the real part in 0th order in the gradient expansion, we can derive:

(A3) Ree−iβ(x)δδφ¯*(x)Γ2′2=Re2e4∫we−iβ(x)+iβ(w)|φ¯(w)| ×PR(x,w)ΔF(w,x)+PF(x,w)ΔA(w,x)eie∫xwAμdzμ+ie∫wxAμdzμ∼2e4|φ¯(x)|∫p[RePR(X,p)F(X,p−e(A−∂β/e)) +PF(X,p)ReGRX,p−e(A−∂β/e)],

with $G\left(w,x\right)\equiv e^{ie\int_{x}^{w}A^{\mu }dz_{\mu }}\Delta \left(w,x\right)$ and $F=\frac{G^{21}+G^{12}}{2}$. This term appears in KG Equations (121) and (123) for $|\bar{\varphi }|$.

We can also derive the following relations:

(A4) Reiφ¯*(x)δδφ¯*(x)Γ2′2=−12∂∂x∫pReΣR′(x,p)∂F(x,p)∂p+ΣF′(x,p)∂ReGR(x,p)∂p+12∫pΣρ′(x,p)iF(x,p)−ΣF′(x,p)ρ(x,p)i+O∂2∂X2,

with the $O(e^{4}|\bar{\varphi }{|}^{2})$ self-energy $\Sigma^{\prime ab}\left(X,p-e(A-\partial \beta /e)\right)=-2e^{4}{\left|\bar{\varphi }\left(X\right)\right|}^{2}P^{ab}\left(X,p\right)$ in 1st order in the gradient expansion. As a result, we can derive:

(A5) Reiφ¯*(X)δδφ¯*(X)Γ2′2−iφ¯(X)δδφ¯(X)Γ2′2=−∂∂X∫pReΣR′(X,p)∂F(X,p)∂p+ΣF′(X,p)∂ReGR(X,p)∂p+∫pΣρ′(X,p)iF(X,p)−ΣF′(X,p)ρ(X,p)i+O∂2∂X2.

In the Hartree–Fock approximation with $O(e^{2})$ and $O(e^{4}|\bar{\varphi }{|}^{2})$ diagrams, since the $O(e^{2})$ interactions never change total charge density in a spatially homogeneous system, the self-energy $\Sigma^{\prime }$ can be replaced by Σ which is written by the sum of $O(e^{2})$ and $O(e^{4}|\bar{\varphi }{|}^{2})$ self-energy. We have used the above relation in Equation (126).

Next we shall write the $O(e^{4}|\bar{\varphi }{|}^{2})$ self-energy ($\Sigma^{\prime }$ and $\Pi^{\prime }$) in the Kadanoff–Baym equations. We can write the self-energy as

(A6) $$\begin{array}{cc} \Sigma^{\prime ab}\left(X,k-e\left(A-\partial \beta /e\right)\right) & =-2e^{4}{\left|\bar{\varphi }\left(X\right)\right|}^{2}\int_{l}D_{ij}^{ab}\left(X,k-l\right)D_{ij}^{ab}\left(X,l\right)\\ & =-2e^{4}{\left|\bar{\varphi }\left(X\right)\right|}^{2}P^{ab}\left(X,k\right),\\ \Pi_{ij}^{\prime ab}\left(X,k\right) & =-4e^{4}{\left|\bar{\varphi }\left(X\right)\right|}^{2}\int_{l}D_{ij}^{ab}\left(X,k-l\right) \end{array}$$

(A7) $$\begin{array}{c} \times \left(G^{ab}\left(X,l-e\left(A-\partial \beta /e\right)\right)+G^{ba}\left(X,-l-e\left(A-\partial \beta /e\right)\right)\right). \end{array}$$

It is possible to prove the H-theorem for the above self-energy in the same way as [58]. Then we find the following term as

(A8) $$\begin{array}{c} e^{4}{\left|\bar{\varphi }\left(X\right)\right|}^{2}\int_{l,p,k}\delta_{l+p-k}\left[D_{T}^{12}\left(l\right)D_{T}^{12}\left(p\right)G^{21}\left(k-e\left(A-\partial \beta /e\right)\right)-D_{T}^{21}\left(l\right)D_{T}^{21}\left(p\right)G^{12}\left(k-e\left(A-\partial \beta /e\right)\right)\right]\\ \times \ln\frac{D_{T}^{12}\left(l\right)D_{T}^{12}\left(p\right)G^{21}\left(k-e\left(A-\partial \beta /e\right)\right)}{D_{T}^{21}\left(l\right)D_{T}^{21}\left(p\right)G^{12}\left(k-e\left(A-\partial \beta /e\right)\right)}\geq 0. \end{array}$$

At the equilibrium state, we find

(A9) $$\begin{array}{cc} f_{T}\left(p\right)=f_{L}\left(p\right)=\frac{1}{\exp\left(\frac{p^{0}}{T}\right)-1}, & f\left(p\right)=\frac{1}{\exp\left(\frac{p^{0}-\mu }{T}\right)-1},\\ \mu =-e\left(A^{0}-\frac{\partial^{0}\beta }{e}\right), \end{array}$$

where *T* is the temperature, μ is the chemical potential, *f* is the distribution function of charged bosons in the Kadanoff–Baym Ansatz, and $f_{T,L}$ is the distribution function of photons in the transverse and longitudinal parts.

Finally let us derive the potential energy $E_{\mathrm{pot},\mathrm{nonl}}$ for $O(e^{4}|\bar{\varphi }{|}^{2})$ self-energy. We can write the Kadanoff–Baym equations with only $O(e^{4}|\bar{\varphi }{|}^{2})$ self-energy in the spatially homogeneous isolated system in $A^{i}-\partial^{i}\beta /e=0$ as

(A10) p2−m2−ReΣR′,F+ReGR,ΣF′−1iFΣρ′−ρΣF′=0,p2−2e2|φ¯|2−ReΠR,T′,FT+ReDR,T,ΠF,T′−1iFTΠρ,T′−ρTΠF,T′=0,p2−2e2|φ¯|2−ReΠR,L′,FL+ReDR,L,ΠF,L′−1iFLΠρ,L′−ρLΠF,L′=0.

As in [75,76], after multiplying $p^{0}$ in this relation and taking the sum, we need to calculate the following integration by $\int_{p}$:

(A11) −∂∂X0∫pReΣR′F+ReGRΣF′−∂∂X0∫pp0∂ReΣR′∂p0F+p0ReGR∂ΣF′∂p0+∫pReΣR′∂F∂X0+∂ReGR∂X0ΣF′−∂∂X012∫pReΠR′ijDFij+ReDRijΠF′ij−12∂∂X0∫pp0∂ReΠR′ij∂p0DFij+p0ReDRij∂ΠF′ij∂p0+12∫pReΠR′ij∂DFij∂X0+∂ReDRij∂X0ΠF′ij−1i∫pp0FΣρ′−ρΣF′−12i∫pp0FijΠρ,ij′−ρijΠF,ij′+∂∂X02∫pp02F+∫pp02DFii=0.

The sum of the 1st and the 4th term on the left-hand side in Equation (A11) is

(A12) $$\begin{array}{cc} \left(1\mathrm{st}+4\mathrm{th}\right) & =-\frac{i}{2}\partial_{0}\int_{p}\left(\Sigma^{\prime 11}G^{11}-\Sigma^{\prime 22}G^{22}\right)-\frac{i}{4}\partial_{0}\int_{p}\left(\Pi_{ij}^{\prime 11}D_{ij}^{11}-\Pi_{ij}^{\prime 22}D_{ij}^{22}\right)\\ & =\frac{i}{2}\partial_{0}[3e^{4}{\left|\bar{\varphi }\right|}^{2}\int_{k,l,p}\delta_{p+k+l}(D_{ij}^{11}\left(p\right)D_{ij}^{11}\left(k\right)\\ & \;\times \left(G^{11}\left(l-e\left(A-\partial \beta /e\right)\right)+G^{11}\left(-l-e\left(A-\partial \beta /e\right)\right)\right)-\left(11\to 22\right))], \end{array}$$

where we have used ReGR=i2G11−G22, ReDR,ij=i2Dij11−Gij22, ReΣR′=i2Σ′11−Σ′22, and ReΠR,ij′=i2Πij′11−Πij′22 with $\Sigma^{\prime aa}\left(k-e\left(A-\partial \beta /e\right)\right)=-2e^{4}{\left|\bar{\varphi }\right|}^{2}\int_{q}D_{ij}^{aa}\left(k-q\right)D_{ij}^{aa}\left(q\right)$ and $\Pi_{ij}^{\prime aa}\left(k\right)=-4e^{4}{\left|\bar{\varphi }\right|}^{2}\int_{l}D_{ij}^{aa}\left(k-l\right)\left(G^{aa}\left(l-e\left(A-\partial \beta /e\right)\right)+G^{aa}\left(-l-e\left(A-\partial \beta /e\right)\right)\right)$. The sum of the 2nd and the 5th term on the left-hand side in Equation (A11) is

(A13) $$\begin{array}{cc} (2\mathrm{nd}+5\mathrm{th})= & \frac{i}{2}\partial_{0}[-e^{4}{\left|\bar{\varphi }\right|}^{2}\int_{k,l,p}\delta_{p+k+l}(D_{ij}^{11}\left(p\right)D_{ij}^{11}\left(k\right)\\ & \times \left(G^{11}\left(l-e\left(A-\partial \beta /e\right)\right)+G^{11}\left(-l-e\left(A-\partial \beta /e\right)\right)\right)-\left(11\to 22\right))]\\ & +\frac{i}{2}\partial_{0}[-e^{4}{\left|\bar{\varphi }\right|}^{2}\int_{k,l,p}\delta_{p+k+l}\;e\left(A^{0}-\frac{\partial^{0}\beta }{e}\right)(\frac{\partial D_{ij}^{11}\left(p\right)}{\partial p^{0}}D_{ij}^{11}\left(k\right)\\ & \times \left(G^{11}\left(-l-e\left(A-\partial \beta /e\right)\right)-G^{11}\left(l-e\left(A-\partial \beta /e\right)\right)\right)-\left(11\to 22\right))], \end{array}$$

where we have used $D_{ij}^{aa}\left(p\right)=D_{ij}^{aa}\left(-p\right)$ with a=1,2. We can write the sum of the 3rd and the 6th term on the left-hand side in Equation (A11) as

(A14) $$\begin{array}{cc} \left(3\mathrm{rd}+6\mathrm{th}\right) & =\frac{i}{2}\int_{k}\left(\Sigma^{\prime 11}\frac{\partial G^{11}}{\partial X^{0}}-\Sigma^{\prime 22}\frac{\partial G^{22}}{\partial X^{0}}\right)+\frac{i}{4}\int_{k}\left(\Pi_{ij}^{\prime 11}\frac{\partial D_{ij}^{11}}{\partial X^{0}}-\Pi_{ij}^{\prime 22}\frac{\partial D_{ij}^{22}}{\partial X^{0}}\right)\\ & =\frac{i}{2}(-2e^{4}|\bar{\varphi }{|}^{2})\int_{k,l}[D_{ij}^{11}\left(k-l+e\left(A-\partial \beta /e\right)\right)D_{ij}^{11}\left(l\right)\frac{\partial G^{11}\left(k\right)}{\partial X^{0}}\\ & \;+D_{ij}^{11}\left(k-l\right)\left(G^{11}\left(l-e\left(A-\partial \beta /e\right)\right)+G^{11}\left(-l-e\left(A-\partial \beta /e\right)\right)\right)\frac{\partial D_{ij}^{11}\left(k\right)}{\partial X^{0}}\\ & \;-(11\to 22)]\\ & =\frac{i}{2}(-2e^{4}|\bar{\varphi }{|}^{2})\int_{k,l,p}[\delta_{k-l-p+e(A-\partial \beta /e)}D_{ij}^{11}\left(p\right)D_{ij}^{11}\left(l\right)\frac{\partial G^{11}\left(k\right)}{\partial X^{0}}\\ & \;+\delta_{k-l-p}D_{ij}^{11}\left(p\right)\left(G^{11}\left(l-e\left(A-\partial \beta /e\right)\right)+G^{11}\left(-l-e\left(A-\partial \beta /e\right)\right)\right)\frac{\partial D_{ij}^{11}\left(k\right)}{\partial X^{0}}\\ & \;-(11\to 22)]\\ & =\frac{i}{2}(-2e^{4}|\bar{\varphi }{|}^{2})\int_{k,l,p}[\frac{1}{2}\delta_{k-l-p+e(A-\partial \beta /e)}D_{ij}^{11}\left(p\right)D_{ij}^{11}\left(l\right)\frac{\partial G^{11}\left(k\right)}{\partial X^{0}}\\ & \;+\frac{1}{2}\delta_{k-l-p-e(A-\partial \beta /e)}D_{ij}^{11}\left(p\right)D_{ij}^{11}\left(l\right)\frac{\partial G^{11}\left(-k\right)}{\partial X^{0}}\\ & \;+\delta_{l-k+p-e(A-\partial \beta /e)}D_{ij}^{11}\left(p\right)G^{11}\left(k\right)\frac{\partial D_{ij}^{11}\left(l\right)}{\partial X^{0}}\\ & \;+\delta_{l-k+p+e(A-\partial \beta /e)}D_{ij}^{11}\left(p\right)G^{11}\left(-k\right)\frac{\partial D_{ij}^{11}\left(l\right)}{\partial X^{0}}-\left(11\to 22\right)], \end{array}$$

and

(A15) $$\begin{array}{cc} (3\mathrm{rd}+6\mathrm{th})= & \frac{i}{2}\partial_{0}[(-e^{4}|\bar{\varphi }{|}^{2})\int_{k,l,p}[\delta_{k-l-p+e(A-\partial \beta /e)}D_{ij}^{11}\left(p\right)D_{ij}^{11}\left(l\right)G^{11}\left(k\right)\\ & +\delta_{k-l-p-e(A-\partial \beta /e)}D_{ij}^{11}\left(p\right)D_{ij}^{11}\left(l\right)G^{11}\left(-k\right)-\left(11\to 22\right)]]\\ & +\frac{i}{2}e^{4}\frac{\partial |\bar{\varphi }{|}^{2}}{\partial X^{0}}\int_{k,l,p}[\delta_{k-l-p+e(A-\partial \beta /e)}D_{ij}^{11}\left(p\right)D_{ij}^{11}\left(l\right)G^{11}\left(k\right)\\ & +\delta_{k-l-p-e(A-\partial \beta /e)}D_{ij}^{11}\left(p\right)D_{ij}^{11}\left(l\right)G^{11}\left(-k\right)-\left(11\to 22\right)]]\\ & +\frac{i}{2}\left(2e^{4}{\left|\bar{\varphi }\right|}^{2}\right)e\left(\partial_{0}\left(A^{0}-\frac{\partial^{0}\beta }{e}\right)\right)\frac{1}{2}\int_{k,l,p}\delta_{k-l-p}[\frac{\partial D_{ij}^{11}\left(p\right)}{\partial p^{0}}D_{ij}^{11}\left(l\right)G^{11}\left(k-e\left(A-\partial \beta /e\right)\right)\\ & -\frac{\partial D_{ij}^{11}\left(p\right)}{\partial p^{0}}D_{ij}^{11}\left(l\right)G^{11}\left(-k-e\left(A-\partial \beta /e\right)\right)-\left(11\to 22\right)]], \end{array}$$

where we have used $D_{ij}^{aa}\left(p\right)=D_{ij}^{aa}\left(-p\right)$ with a=1,2 and

(A16) $$\begin{array}{ccc} \frac{\partial }{\partial X^{0}}\delta_{k-l-p+e(A-\partial \beta /e)} & = & -e\frac{\partial \left(A^{0}-\frac{\partial^{0}\beta }{e}\right)}{\partial X^{0}}\frac{\partial }{\partial p^{0}}\delta_{k-l-p+e(A-\partial \beta /e)}, \end{array}$$

(A17) $$\begin{array}{ccc} \frac{\partial }{\partial X^{0}}\delta_{k-l-p-e(A-\partial \beta /e)} & = & e\frac{\partial \left(A^{0}-\frac{\partial^{0}\beta }{e}\right)}{\partial X^{0}}\frac{\partial }{\partial p^{0}}\delta_{k-l-p-e(A-\partial \beta /e)}. \end{array}$$

We find that the 1st term in Equation (A13) and the 1st term in Equation (A15) has the same form as Equation (A12) except constant factors. As a result, Equation (A11) is rewritten as

(A18) +∂∂X02∫pp02F+∫pp02DFii−13∂0∫pReΣR′F+ReGRΣF′−16∂0∫pReΠR′ijDFij+ReDRijΠF′ij−1i∫pp0FΣρ′−ρΣF′−12i∫pp0FijΠρ,ij′−ρijΠF,ij′+i2∂0[−e4|φ¯|2∫k,l,pδp+k+leA0−∂0βe(∂Dij11(p)∂p0Dij11(k)×G11(−l−e(A−∂β/e))−G11(l−e(A−∂β/e))−(11→22))]+i2e4∂|φ¯|2∂X0∫k,l,p[δk−l−p+e(A−∂β/e)Dij11(p)Dij11(l)G11(k)+δk−l−p−e(A−∂β/e)Dij11(p)Dij11(l)G11(−k)−(11→22)]]+i22e4|φ¯|2e∂0A0−∂0βe12∫k,l,pδk−l−p[∂Dij11(p)∂p0Dij11(l)G11(k−e(A−∂β/e))−∂Dij11(p)∂p0Dij11(l)G11(−k−e(A−∂β/e))−(11→22)]]=0.

We find that 4th, 5th, ..., 8th terms cancel with energy terms from Klein–Gordon equations. By multiplying the Klein–Gordon equations in Equation (121) by $2\partial_{0}\left|\bar{\varphi }\right|$, we find that the following terms appear in both the isolated system and the open systems:

(A19) −2e4∂|φ¯|2∂X0∫kRePR(k)F(k−e(A−∂β/e))+PF(k)ReGR(k−e(A−∂β/e))+eA0−∂0βe∂∂X0∫pReΣR′∂F∂p0+ΣF′∂ReGR∂p0−1ieA0−∂0βe∫pFΣρ′−ρΣF′,

where we have used the relations (127) and (128) with the integration of the KB equations of charged bosons in Equations (109) and (113). The 1st term in Equation (A19) cancels with the 7th term in Equation (A18). The 2nd term in Equation (A19) cancels with the 6th and the 8th term in Equation (A18). For the 3rd term in Equation (A19), 4th and 5th terms in Equation (A18), we can also show that

(A20) $$\begin{array}{ccc} -\frac{1}{i}\int_{p}p^{0}\left(F\Sigma_{\rho }^{\prime }-\rho \Sigma_{F}^{\prime }\right)-\frac{1}{2i}\int_{p}p^{0}\left(F_{ij}\Pi_{\rho ,ij}^{\prime }-\rho_{ij}\Pi_{F,ij}^{\prime }\right) & \\ -\frac{1}{i}e\left(A^{0}-\frac{\partial^{0}\beta }{e}\right)\int_{p}\left(F\Sigma_{\rho }^{\prime }-\rho \Sigma_{F}^{\prime }\right) & = & 0, \end{array}$$

for $O(e^{4}|\bar{\varphi }{|}^{2})$ self-energy. Therefore we arrive at Equation (137). There is no memory integral term in the total energy in QED in open systems in the Hartree–Fock approximation with the LO approximation in the tunneling variable and coupling expansion in the 1st order in the gradient expansion. (The memory integral term in the energy in [58] appears in neglecting NLO $O(e^{4})$ contributions in $\frac{\Gamma_{2}^{\prime }}{2}$.)
